# Early Restoration of *Shank3* Expression in *Shank3* Knock-Out Mice Prevents Core ASD-Like Behavioral Phenotypes

**DOI:** 10.1523/ENEURO.0332-19.2020

**Published:** 2020-06-01

**Authors:** Thomas C. Jaramillo, Zhong Xuan, Jeremy M. Reimers, Christine O. Escamilla, Shunan Liu, Craig M. Powell

**Affiliations:** 1 Department of Neurology and Neurotherapeutics, University of Texas Southwestern Medical Center, Dallas, TX 75390-8813; 2 Department of Neurobiology, University of Alabama at Birmingham, Birmingham, AL; 3 Civitan International Research Center at UAB, Birmingham, AL 35233

**Keywords:** autism, autism spectrum disorder, behavior, genetic reversal, Phelan–McDermid syndrome, Shank3

## Abstract

Several genes are associated with increased risk for autism spectrum disorder (ASD), neurodevelopmental disorders that present with repetitive movements and restricted interests along with deficits in social interaction/communication. While genetic alterations associated with ASD are present early in life, ASD-like behaviors are difficult to detect in early infancy. This raises the issue of whether reversal of an ASD-associated genetic alteration early in life can prevent the onset of ASD-like behaviors. Genetic alterations of *SHANK3*, a well-characterized gene encoding a postsynaptic scaffolding protein, are estimated to contribute to ∼0.5% of ASD and remain one of the more replicated and well-characterized genetic defects in ASD. Here, we investigate whether early genetic reversal of a *Shank3* mutation can prevent the onset of ASD-like behaviors in a mouse model. Previously, we have demonstrated that mice deficient in *Shank3* display a wide range of behavioral abnormalities such as repetitive grooming, social deficits, anxiety, and motor abnormalities. In this study, we replicate many of these behaviors in *Shank3* mutant mice. With early genetic restoration of wild-type (WT) *Shank3*, we rescue behaviors including repetitive grooming and social, locomotor, and rearing deficits. Our findings support the idea that the underlying mechanisms involving ASD behaviors in mice deficient in *Shank3* are susceptible to early genetic correction of *Shank3* mutations.

## Significance Statement

Rare, *de novo*, single gene copy number variants and mutations are known causes of autism. The *SHANK3* gene is among the most common and replicated genetic causes of autism. With the advent of gene therapy, interest is growing in understanding whether genetic animal models of autism can have their phenotypes ameliorated by genetic reversal. This study confirms that early genetic restoration of a *Shank3* mutant mouse model can ameliorate behavioral symptoms with face validity for autism.

## Introduction

The *Shank3* gene encodes a multi-domain, scaffolding protein located at the postsynaptic density of excitatory synapses that interacts with a number of scaffolding and signaling proteins to form complexes that ensure proper synaptic formation and function (Naisbitt et al., 1999; Tu et al., 1999; Ebert and Greenberg, 2013). Approximately 0.5–1% of all individuals with autism spectrum disorder (ASD) have *SHANK3* mutations (Jiang and Ehlers, 2013). Loss-of-function of one copy of *SHANK3* is a known cause of Phelan–McDermid syndrome (PMS), a cause of ASD and intellectual disability (Phelan, 2008; Costales and Kolevzon, 2015; Tachibana et al., 2017; Mitz et al., 2018). ASD is characterized by the presentation of two core phenotypes, deficits in social interaction/communication and restricted interests and repetitive behaviors. In patients, symptoms of ASD can be evident as early as 12 months, while actual diagnosis of these symptoms as ASD may not occur until later in childhood. In this study we address the possibility of early genetic intervention to prevent the onset of ASD-like behaviors.

Previously, we generated and characterized a mouse model deficient in a subset of major isoforms of *Shank3* (Shank3^E13^) that behaviorally displayed excessive grooming, social interaction deficits, decreased rearing, locomotor deficits, and learning and memory deficits (Jaramillo et al., 2017). Additionally, other studies in similar *Shank3* mouse models have replicated these findings in addition to observing decreased hippocampal long-term potentiation, morphologic and structural abnormalities like reduced spine density and longer dendritic length, along with a reduction in synaptic proteins that associate with SHANK3 (Bozdagi et al., 2010; Peça et al., 2011; Wang et al., 2011; Yang et al., 2012; Kouser et al., 2013). One elegant study also examined the ability of adult and early developmental genetic reversal to rescue phenotypes in a *Shank3* mutant mouse model (Mei et al., 2016). In efforts parallel to those of Guoping Feng’s laboratory (Mei et al., 2016), we also generated a similar, reversible *Shank3* mouse model to examine the question of developmental reversibility of phenotypes.

Our Shank3^E13^ mouse model was generated by inserting a transcriptional “stop” (neo-stop) cassette flanked by loxP sites into the intron before the start of exon 13, the first exon encoding the PDZ domain [postsynaptic density-95 (PSD-95)/disk large (DLG)/zona occludens-1 (ZO-1)]. Because of the flanking loxP sites, the Shank3^E13^ model can be genetically reversed to wild-type (WT) by removal of the neo-stop cassette on expression of Cre-recombinase. Our model is similar to the one generated in Feng’s laboratory in that both induce *Shank3* gene expression at its endogenous genomic locus ensuring that SHANK3 expression is within its physiologic concentrations. However, while Feng’s model uses tamoxifen for *Shank3* induction which leads to some toxicity including weight loss in WT mice, we used a 2-transgene system involving TTA and Cre expression in an attempt to temporally regulate *Shank3* expression. Two transgenic mouse lines, tetracycline-controlled activator protein under control of the neuron-specific enolase promoter (NSE-tTA) and Cre recombinase under the control of a tetracycline-response promoter element (tetO; tetO-Cre) were bred with the Shank3^E13^ mutant mouse model. The NSE-tTA transgene leads to expression of tTA at approximately embryonic day (E)18 in mice (Forss-Petter et al., 1990; Alouani et al., 1993). tTA expression activates the tetO promoter to drive Cre-recombinase expression, allowing for recombination of the premature stop cassette between the flanking loxP sites (i.e., genetic reversal). Previous reversal studies in *Shank3* mouse models of ASD (Mei et al., 2016) focused on restoration of SHANK3 expression in homozygous knock-out (KO) mice. In this study, however, we include the heterozygous Shank3 mice to provide more accurate construct validity to the human condition. While we had planned to examine both adult and early genetic restoration, we are unable to prevent restoration of Shank3 using doxycycline in our model. Following early genetic reversal, Shank3^E13^ mutant mice express WT *Shank3* and no longer display repetitive grooming or social deficits. As a control and replication of our previous findings, these atypical behaviors were observed in Shank3^E13^ mutant mice expressing only one of the two transgenes. These results replicate many, but not all, of our original findings in the Shank3^E13^ mutant mice and confirm that some behaviors with face validity for ASD remain responsive to re-expression of WT *Shank3* early in development. Our findings suggest early genetic reversal as a potential treatment for ASD-like behaviors associated with *Shank3* deficiency.

## Materials and Methods

### Generation of genetically reversible Shank3^E13^ mutant mice

The generation of the Shank3^E13^ mutant mice is described in our previous publication (Jaramillo et al., 2017). Briefly, a neo-stop targeting construct was designed by combining PGK-neo gene cassette with the His3-SV40 pA sequences and inserted into a unique BglII site located within intron 12, after exon 12 of the mouse *Shank3* gene (Dragatsis and Zeitlin, 2001; Guy et al., 2007). The neo-stop cassette prevents transcription of *Shank3* beyond exon 12. The neo-stop cassette is flanked by loxP sites making this insertion reversible in the presence of cre-recombinase. Following verification of proper insertion, Shank3^E13^ mice were used to generate two lines of mice.

**Figure 1.**
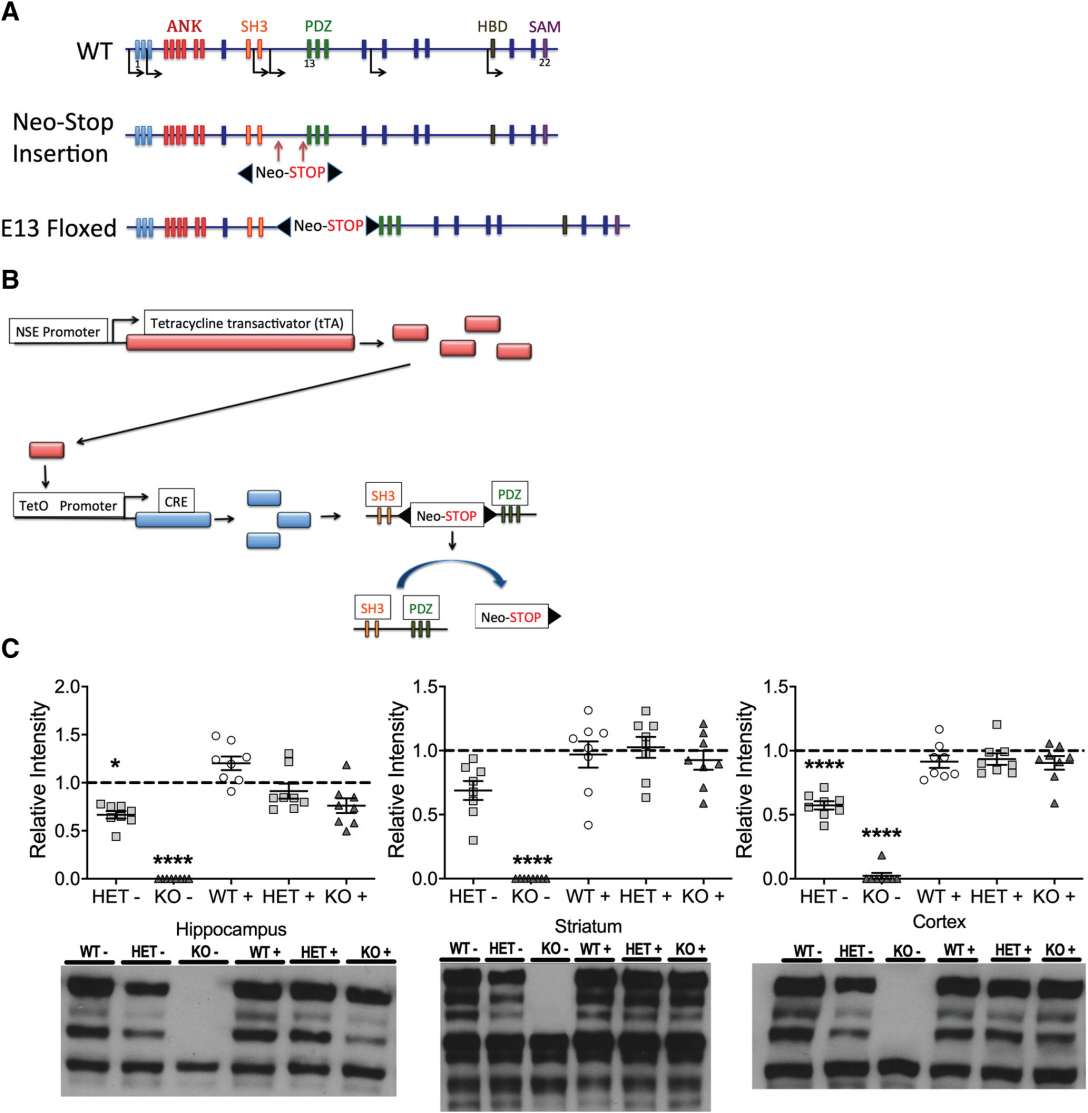
Generation of genetically reversible *Shank3* mutant mice. ***A***, top, A diagram of *Shank3* gene along with its 22 exons (colored bars), six known promoters (arrows), and its five conserved domains, Ankyrin repeat domain (ANK), Src homology 3 (SH3) domain, PDZ domain, a proline-rich region containing homer- and cortactin-binding sites (HBD), and a sterile α motif (SAM) domain. Middle, Insertion of the neo-stop cassette flanked by loxP sites (black arrowheads) into intron 12 just before exon 13 of the *Shank3* gene. Bottom, The generation of the Shank3^FloxE13^ following insertion of the neo-stop cassette. ***B***, Regulation of *Shank3* gene expression. Top, Schematic representation of the NSE-tTA transgene. Following NSE promoter activation, tTA expression binds the tetO promoter and thus drives Cre expression. Bottom, Cre protein binds to the loxP sites flanking the regulatory cassette and, following recombination, excision of the neo-STOP results in restoration of WT *Shank3* gene sequence. ***C***, Biochemical analysis in whole lysate preps from hippocampus, striatum, and cortical tissue from both genetically reversed Shank3^E13^ mice (WT+, HET+, KO+) and Shank3^E13^ control mice (HET– and KO–; WT– is represented by the dash line); *p < 0.05; ****p < 0.0001; *n* = 8 WT–, *n* = 8 HET–, *n* = 8 KO–, *n* = 8 WT+, *n* = 8 HET+, *n* = 8 KO+.

One line consisted of heterozygous Shank3^E13^ mice bred with NSE-tTA (tetracycline-regulated activator protein under the control of neuron specific enolase promoter; The Jackson Laboratory stock #003767). Although the description for the NSE-tTA mouse line from The Jackson Laboratory and a publication (Chen et al., 1998) suggest selective expression of tTA in the striatum and cerebellum, our studies revealed global expression of tTA under our specific conditions (Fig. 1*C*). To determine whether a mouse was positive for the NSE-tTA transgene, two sets of primers were used. Internal primers for the transgene, forward 5′-CAAATGTTGCTTGTCTGGTG-3′ and reverse 5′-GTCAGTCGAGTGCACAGTTT-3′, produced a 200-bp band. Primers for the transgene, forward 5′-CGCTGTGGGGCATTTTACTTTAG-3′ and reverse 5′-CATGTCCAGATCGAAATCGTC-3′ produced a 450-bp band for a second confirmation.

A second mouse line consisted of heterozygous Shank3^E13^ mice bred with tetO-Cre (cre-recombinase under control of a tetracycline-response promoter element, tetO; The Jackson Laboratory stock #003767). To verify the presence of the transgene, forward 5′-CGCTGTGGGGCATTTTACTTTAG-3′ and reverse 5′-CATGTCCAGATCGAAATCGTC-3′ primers produced a 100-bp band, confirming presence of the tetO-Cre transgene. A pair of internal primers was also used; forward 5′-CAAATGTTGCTTGTCTGGTG-3′ and reverse 5′-GTCAGTCGAGTGCACAGTTT-3′ produced a 200-bp band. Finally, the two mouse lines generated (Shank3^E13/NSE-tTA^ mice and Shank3^E13/tetO-Cre^ mice) were bred together to generate Shank3^E13/NSE-tTA/tetO-Cre^ mice (Shank3+) with both the tTA and tetO-cre transgenes and either WT, heterozygous, or homozygous for the *Shank3* ^E13^ allele. Mice that only retained one of the transgenes were used as controls (*Shank3*–) and remained mutant for *Shank3*. It is worth noting that our attempts to use doxycycline to suppress cre-recombinase expression in this particular model were unsuccessful. Thus, we focused on the early developmental rescue of *Shank3* gene expression in the absence of doxycycline.

### Behavioral overview

All mice tested were age- and sex-matched littermate progeny of matings between mice heterozygous for Shank3^E13^ with floxed neo-stop cassette and one of the regulator transgenes, NSE-tTA or tetO-cre. Shank3^E13^ with both regulator transgenes constituted the experimental cohort and are referred to as Shank3+ mice, while Shank3^E13^ mice with only one of the two regulator transgenes constituted the control cohort and are referred to as *Shank3*– (WT–, HET–, KO–). Genetic background is consistent among all mice due to the use of a single breeding strategy to produce all experimental offspring from the same cross. An experimenter blind to genotypes performed all behavioral tests. The behavioral testing consisted of two cohorts. The first included mice WT+, heterozygous+ (HET+), and homozygous+ (KO+) for floxed, neo-stop-cassette-containing Shank3^E13^ expressing both transgenes (tetOp-cre and NSE-tTA). The second cohort included WT–, HET–, and KO– Shank3^E13^ mice with only one of the two transgenes (tetO-cre or NSE-tTA). Cohort 1 consisted of *n* = 22 WT (WT+, 10 male and 12 female), *n* = 17 heterozygous (HET+, eight males and nine females), *n* = 14 KO (KO+, eight males and six females) mice, while cohort 2 consisted of *n* = 27 WT (WT–, 16 male and 11 female), *n* = 20 heterozygous (HET–, 10 males and 10 females), *n* = 19 KO (KO–, 10 males and 9 females) mice.

Behaviors were tested in the following order: elevated plus maze, dark/light, open field (OF), locomotor, rearing, grooming, three-box social interaction, caged conspecific (also called social interaction with a caged adult), marble burying, rotarod, social interaction with free moving juvenile mouse, genotype-sex-matched adult social interaction, olfactory “cookie-finding” palatable treat test, nesting, cue and contextual fear conditioning, and Morris water maze. Behavioral results are presented in a different order than tested to ease the flow of presentation. Analysis of behavioral data were conducted using StatPlus software (version 2015, AnalystSoft) using either two-way ANOVA or three-way repeated measures ANOVA with genotype and sex as the main variables and trial, bouts, or time as the repeated measure where applicable. *Post hoc* planned comparisons were applied for significant effects and interactions. For detailed statistical results, see Table 1.

**Table 1:** Statistical analysis

Shank3– (*n* = 11 WT, 8 HET, 7 KO pairs)	Genotype sex match	Interactiontime (s)	Genotype and sex *Post hoc*: Scheffe genotype Fig. 2*A*	Two-way ANOVA; no main effect of sex: *F*_(1,28)_ = 0.935, *p* = 0.343; **main effect of genotype: *F*_(2,28)_ = 5.338, *p* < 0.012;** no main effect of sex × genotype interaction: *F*_(1,28)_ = 2.932, *p* = 0.073 **HET vs KO: *p* < 0.021** HET vs WT: *p* = 0.750 KO vs WT: *p* = 0.0508
Shank3+ (*n* = 11 WT, 8 HET, 7 KO pairs)		Interactiontime (s)	Genotype and sex Fig. 2*B*	Two-way ANOVA; no main effect of sex: *F*_(1,25)_ = 0.733, *p* = 0.401; no main effect of genotype: *F*_(2,25)_ = 0.098, *p* = 0.906; no main effect of sex × genotype interaction: *F*_(1,25)_ = 0.517, *p* = 0.604
Shank3– (*n* = 24 WT, 16 HET, 20 KO)	Social interaction with a juvenile	Interactiontime (s)	Sex, genotype, trial *Post hoc*: pairwise *t* test: initial vs recognition Fig. 2*C*	Three-way ANOVA; no main effect of sex: *F*_(1,119)_ = 1.591, *p* = 0.209; **main effect of genotype: *F*_(2,119)_ = 3.725, *p* < 0.027; main effect of trial: *F*_(1,119)_ = 11.937, *p* < 0.0007**; no main effect of sex × genotype interaction: *F*_(2,119)_ = 0.229, *p* = 0.795; no main effect of sex × trial: *F*_(1,119)_ = 2.984, *p* = 0.086; no main effect of genotype × trial: *F*_(1,119)_ = 2.261, *p* = 0.1088; **main effect of genotype × sex × target: *F*_(2,119)_ = 2.761, *p* < 0.034** **WT: *p* < 0.002** HET: *p* = 0.317 KO: *p* = 0.373
Shank3+ (*n* = 24 WT, 19 HET, 16 KO)		Interactiontime (s)	Sex, genotype, trial *Post hoc*: pairwise *t* test: initial vs recognition Fig. 2*D*	Three-way ANOVA; no main effect of sex: *F*_(1,121)_ = 1.70, *p* = 0.193; no main effect of genotype: *F*_(2,121)_ = 0.275, *p* = 0.759; main effect of trial: *F*_(1,121)_ = 16.421, *p* < 0.00009; no main effect of sex × genotype interaction: *F*_(2,121)_ = 0.568, *p* = 0.568; **main effect of sex × trial: *F*_(1,121)_ = 0.049, *p* = 0.951; main effect of genotype × trial: *F*_(1,121)_ = 0.049,*p* = 0.951; main effect of genotype × sex × target: *F*_(2,121)_ = 6.527,*p* < 0.002** **WT: *p* < 0.024** **HET: *p* < 0.04** **KO: *p* < 0.019**
Shank3– (*n* = 24 WT, 16 HET, 20 KO)		Trial 1: chamber bias	Genotype and chamber *Post hoc*: Scheffe; sex × chamber Fig. 2*E*	Three-way ANOVA; no main effect of sex: *F*_(1,126)_ = 0.539, *p* = 0.464; no main effect of genotype: *F*_(2,126)_ = 0.115, *p* = 0.890; no main effect of chamber: *F*_(1,126)_ = 0.362, *p* = 0.548; no main effect of sex × genotype interaction: *F*_(2,126)_ = 0.676, *p* = 0.510; **main effect of sex × chamber: *F*_(1,126)_ = 16.42, *p* < 0.00009**; **main effect of genotype × chamber: *F*_(1,126)_ =, *p* < 0.043**; no main effect of genotype × sex × chamber: *F*_(2,126)_ = 0.676, *p* = 0.510 Back: *p* = 0.117 Front: *p* = 0.294
		Trial 2: duration in chamber; inanimate vs social	Genotype and interaction target (social vs inanimate) *Post hoc*: Scheffe chamber: mouse vs inanimate Sex × chamber Fig. 2*F*	Three-way ANOVA; main effect of sex: *F*_(1,127)_ = 0.469, *p* = 0.494; main effect of genotype: *F*_(2,127)_ = 0.828, *p* = 0.439; **main effect of chamber: *F*_(1,127)_ = 37.75, *p* < 0.00001**; main effect of sex × genotype interaction: *F*_(2,127)_ = 0.131, *p* = 0.877; **main effect of sex × chamber: *F*_(1,27)_ = 7.60, *p* < 0.006**; main effect of genotype × chamber: *F*_(1,127)_ = 2.27, *p* < 0.106; main effect of genotype × sex × chamber: *F*_(2,127)_ = 1.51, *p* = 0.223 **WT: *p* < 0.00000003** HET: *p* = 0.196 **KO: *p* < 0.0012** Female: inanimate vs mouse *p* = 0.233 **Male: inanimate vs mouse *p* < 0.0000001**
		Trial 3: duration in chamber; familiar vs novel	Sex, genotype, and chamber *Post hoc* Scheffe chamber: novel vs familiar Fig. 2*G*	Three-way ANOVA; main effect of sex: *F*_(1,122)_ = 0.472, *p* = 0.493; main effect of genotype: *F*_(2,122)_ = 0.150, *p* = 0.860; **main effect of chamber: *F*_(1,122)_ = 2.242, *p* < 0.0137**; main effect of sex × genotype interaction: *F*_(2,122)_ = 0.070, *p* = 0.924; main effect of sex × chamber: *F*_(1,122)_ = 3.54, *p* = 0.062; main effect of genotype × chamber: *F*_(1,122)_ = 2.816, *p* = 0.064; **main effect of genotype × sex × chamber:*F*_(2,122)_ = 0.049, *p* = 0.951** **WT: *p* < 0.003** HET: *p* = 0.439 KO: *p* = 0.442
Shank3+ (*n* = 24 WT, 19 HET, 20 KO)	3-Box social interaction	Trial 1: chamber bias	Genotype and chamber Fig. 2*H*	Three-way ANOVA; main effect of sex: *F*_(1,81)_ = 3.306, *p* = 0.073; main effect of genotype: *F*_(2,81)_ = 0.071, *p* = 0.930; main effect of chamber: *F*_(1,81)_ = 0.059, *p* = 0.808; main effect of sex × genotype interaction: *F*_(2,81)_ = 0.134, *p* = 0.874; main effect of sex × chamber: *F*_(1,81)_ = 0.001, *p* = 0.965; main effect of genotype × chamber: *F*_(1,81)_ =,*p* = 0.042; main effect of genotype × sex × chamber: *F*_(2,81)_ = 0.784,*p* = 0.460
		Trial 2: duration in chamber; inanimate vs social	Genotype and interaction target (social vs inanimate) *Post hoc*: Scheffe; chamber: mouse vs inanimate Fig. 2*I*	Three-way ANOVA; no main effect of sex: *F*_(1,87)_ = 0.313, *p* = 0.577; no main effect of genotype: *F*_(2,87)_ = 1.121, *p* = 0.300; **main effect of chamber: *F*_(1,87)_ = 15.327, *p* < 0.0002**; no main effect of sex × genotype interaction: *F*_(2,87)_ = 0.067, *p* = 0.934; no main effect of sex × chamber: *F*_(1,87)_ = 0.940, *p* = 0.335; no main effect of genotype × chamber: *F*_(1,87)_ = 0.446, *p* = 0.641; no main effect of genotype × sex × chamber: *F*_(2,87)_ = 2.049, *p* = 0.135 **WT: *p* < 0.0044** HET: *p* = 0.153 **KO: *p* < 0.0012**
		Trial 3: duration in chamber; familiar vs novel	Sex, genotype, and chamber *Post hoc*: Scheffe; chamber: novel vs familiar Fig. 2*J*	Three-way ANOVA; no main effect of sex: *F*_(1,85)_ = 0.175, *p* = 0.676; no main effect of genotype: *F*_(2,85)_ = 0.354, *p* = 0.703; **main effect of chamber: *F*_(1,85)_ = 14.75, *p* < 0.0002;** no main effect of sex × genotype interaction: *F*_(2,85)_ = 0.803, *p* = 0.451; no main effect of sex × chamber: *F*_(1,85)_ = 0.0021, *p* = 0.963; no main effect of genotype × chamber: *F*_(1,85)_ = 0.451, *p* = 0.638; **main effect of genotype × sex × chamber: *F*_(2,85)_ = 4.321, *p* < 0.016** **WT: *p* < 0.017** HET: *p* = 0.099 **KO: *p* < 0.037**
Shank3– (*n* = 24 WT, 16 HET, 20 KO)	Caged conspecific	Interaction time	Sex, genotype, target Fig. 2*K*	Three-way ANOVA; no main effect of sex: *F*_(1,122)_ = 0.720, *p* = 0.397; no main effect of genotype: *F*_(2,122)_ = 0.652, *p* = 0.522; **main effect of target: *F*_(1,122)_ = 26.610, *p* < 1.09E-6**; no main effect of sex × genotype interaction: *F*_(2,122)_ = 0.218, *p* = 0.804; no main effect of sex × target: *F*_(1,122)_ = 1.029, *p* = 0.312; no main effect of genotype × target:*F*_(1,122)_ = 0.759, *p* = 0.470; no main effect of genotype × sex ×target: *F*_(2,122)_ = 1.730, *p* = 0.182
Shank3+ (*n* = 24 WT, 19 HET, 16 KO)		Interaction time	Sex, genotype, target Fig. 2*L*	Three-way ANOVA; no main effect of sex: *F*_(1,107)_ = 0.004, *p* = 0.945; no main effect of genotype: *F*_(2,107)_ = 0.785, *p* = 0.458; **main effect of target: *F*_(1,107)_ = 6.554, *p* < 0.012**; no main effect of sex × genotype interaction: *F*_(2,107)_ = 0.042, *p* = 0.958; no main effect of sex × target: *F*_(1,107)_ = 0.737, *p* = 0.392; no main effect of genotype × target:*F*_(1,107)_ = 0.475, *p* = 0.623; no main effect of genotype × sex ×target: *F*_(2,107)_ = 0.085, *p* = 0.918
Shank3– (*n* = 24 WT, 16 HET, 20 KO)	Grooming	Duration	Sex and genotype *Post hoc*: Scheffe Fig. 3*A*	Two-way ANOVA; no main effect of sex: *F*_(1,62)_ = 1.638, *p* = 0.205; **main effect of genotype: *F*_(2,62)_ = 11.844, *p* < 0.00005**; no sex × genotype interaction: *F*_(1,62)_ = 1.347, *p* = 0.267 **WT vs HET: *p* < 0.00045** HET vs KO: *p* = 0.968 **WT vs KO: *p* < 0.0007**
		Bouts	Sex and genotype *Post hoc*: Scheffe Fig. 3*B*	Two-way ANOVA; **main effect of sex: *F*_(1,62)_ = 8.226, *p* < 0.0057; main effect of genotype: *F*_(2,62)_ = 3.730, *p* < 0.030**; no main effect of sex × genotype interaction: *F*_(1,62)_ = 0.750, *p* = 0.476 WT vs HET: *p* = 0.666 HET vs KO: *p* = 0.265 **WT vs KO: *p* < 0.030**
Shank3+ (*n* = 24 WT, 19 HET, 16 KO)		Duration	Sex and genotype Fig. 3*C*	Two-way ANOVA; no main effect of sex: *F*_(1,60)_ = 0.015, *p* = 0.901; no main effect of genotype: *F*_(2,60)_ = 0.558, *p* = 0.575; no main effect of sex × genotype interaction: *F*_(1,60)_ = 1.15, *p* = 0.323
		Bouts	Sex and genotype Fig. 3*D*	Two-way ANOVA; no main effect of sex: *F*_(1,60)_ = 0.0975, *p* = 0.755; no main effect of genotype: *F*_(2,60)_ = 0.398, *p* = 0.673; no main effect of sex × genotype interaction: *F*_(1,60)_ = 0.514, *p* = 0.600
Shank3– (*n* = 24 WT, 16 HET, 20 KO)	Open field	Time in left	Sex and genotype Fig. 3*E*	Two-way ANOVA; no main effect of sex: *F*_(1,65)_ = 0.619, *p* = 0.434; main effect of genotype: *F*_(2,65)_ = 1.336, *p* = 0.270; no main effect of sex × genotype interaction: *F*_(1,65)_ = 0.177, *p* = 0.838
		Distance traveled left	Sex and genotype Fig. 3*F*	Two-way ANOVA; no main effect of sex: *F*_(1,65)_ = 0.764, *p* = 0.385; main effect of genotype: *F*_(2,65)_ = 3.12, *p* = 0.051; sex × genotype interaction: *F*_(1,65)_ = 0.589, *p* = 0.557
		Thigmotaxis	Sex and genotype Fig. 3*F*	Two-way ANOVA; no main effect of sex: *F*_(1,65)_ = 0.986, *p* = 0.324; main effect of genotype: *F*_(2,65)_ = 2.59, *p* = 0.083; sex × genotype interaction: *F*_(1,65)_ = 0.124, *p* = 0.882
Shank3+ (*n* = 24 WT, 19 HET, 16 KO)		Time in left	Sex and genotype *Post hoc*: Scheffe Sex: female Sex: male Male vs female Fig. 3*G*	Two-way ANOVA; no main effect of sex: *F*_(1,46)_ = 3.477, *p* < 0.0402; **main effect of genotype: *F*_(2,46)_ = 6.864, *p* < 0.012**; no main effect of sex × genotype interaction: *F*_(1,46)_ = 2.137, *p* = 0.130 **WT vs HET: *p* < 0.014** HET vs KO: *p* = 0.067 WT vs KO: *p* = 0.983 WT vs HET: *p* = 0.973 HET vs KO: *p* = 0.984 WT vs KO: *p* = 0.911 WT: *p* = 0.710 **HET: *p* < 0.001** KO: *p* = 0.864
		Distance traveled left	Sex and genotype Male vs female Fig. 3*H*	Two-way ANOVA; no main effect of sex: *F*_(1,46)_ = 0.082, *p* = 0.920; **main effect of genotype: *F*_(2,46)_ = 17.00, *p* < 0.0001**; no main effect of sex × genotype interaction: *F*_(1,46)_ = 0.012, *p* = 0.987 **WT: *p* < 0.020** **HET: *p* < 0.015** **KO: *p* < 0.027**
		Thigmotaxis	Sex and genotype Male vs female Fig. 3*H*	Two-way ANOVA; no main effect of sex: *F*_(1,46)_ = 0.038, *p* = 0.961; **main effect of genotype: *F*_(2,46)_ = 6.729, *p* < 0.012**; no main effect of sex × genotype interaction: *F*_(1,46)_ = 0.162, *p* = 0.850 WT: *p* = 0.199 HET: *p* = 0.223 KO: *p* = 0.054
Shank3– (*n* = 24 WT, 16 HET, 20 KO)	Dark-light test	Latency to explore	Latency to explore Fig. 3*I*	Two-way ANOVA; no main effect of sex: *F*_(1,50)_ = 2.37, *p* = 0.130; no main effect of genotype: *F*_(2,50)_ = 0.128, *p* = 0.879; no main effect of sex × genotype interaction: *F*_(1,50)_ = 0.1.27, *p* = 0.288
		Duration in dark chamber	Sex and genotype *Post hoc*: Scheffe Sex: female Sex: male Female vs male Fig. 3*J*	Two-way ANOVA; no main effect of sex: *F*_(1,50)_ = 0.976, *p* = 0.328; **main effect of genotype: *F*_(2,50)_ = 3.70, *p* < 0.032; main effect of sex × genotype interaction: *F*_(1,50)_ = 3.741, *p* < 0.031** **HET vs KO: *p* < 0.032** HET vs WT: *p* = 0.300 KO vs WT: *p* = 0.395 **HET vs KO: *p* < 0.001** HET vs WT: *p* = 0.215 KO vs WT: *p* = 0.078 HET vs KO: *p* = 0.997 HET vs WT: *p* = 0.990 KO vs WT: *p* = 0.998 WT: *p* = 0.067 **HET: *p* < 0.035** KO: *p* = 0.584
		Duration in light chamber	Sex and genotype *Post hoc*: Scheffe Sex: female Sex: male Female vs male Fig. 3*J*	Two-way ANOVA; no main effect of sex: *F*_(1,50)_ = 0.976, *p* = 0.328; **main effect of genotype: *F*_(2,50)_ = 3.70, *p* < 0.032**; **sex × genotype interaction: *F*_(1,50)_ = 3.741, *p* < 0.031** **HET vs KO: *p* < 0.032** HET vs WT: *p* = 0.300 KO vs WT: *p* = 0.395 **HET vs KO: *p* < 0.0015** HET vs WT: *p* = 0.194 KO vs WT: *p* = 0.082 HET vs KO: *p* = 0.997 HET vs WT: *p* = 0.990 KO vs WT: *p* = 0.998 WT: *p* = 0.584 **HET: *p* < 0.035** KO: *p* = 0.067
Shank3+ (*n* = 24 WT,19 HET, 16 KO)	Dark-light test	Latency to explore	Sex and genotype Fig. 3*K*	Two-way ANOVA; no main effect of sex: *F*_(1,47)_ = 0.649, *p* = 0.424; no main effect of genotype: *F*_(2,47)_ = 0.812, *p* = 0.450; no main effect of sex × genotype interaction: *F*_(1,47)_ = 0.396, *p* = 0.674
		Duration in dark chamber	Sex and genotype *Post hoc*: Scheffe Female vs male Female Male Fig. 3*L*	Two-way ANOVA; no main effect of sex: *F*_(1,47)_ = 1.405, *p* = 0.242; no main effect of genotype: *F*_(2,47)_ = 0.177, *p* = 0.838; no main effect of sex × genotype interaction: *F*_(1,47)_ = 0.183, *p* = 0.833 WT: *p* = 0.511 HET: *p* = 0.862 KO: *p* = 0.259 HET vs KO: *p* = 0.997 HET vs WT:0.995 KO vs WT: *p* = 0.999 HET vs KO: *p* = 0.712 HET vs WT: *p* = 0.939 KO vs WT: *p* = 0.872
		Duration in light chamber	Sex and genotype Fig. 3*L*	Two-way ANOVA; no main effect of sex: *F*_(1,47)_ = 1.40, *p* = 0.242; no main effect of genotype: *F*_(2,47)_ = 0.177, *p* = 0.838; no main effect of sex × genotype interaction: *F*_(1,47)_ = 0.183, *p* = 0.833
Shank3– (*n* = 24 WT, 16 HET, 20 KO)	Elevated plus maze	Duration in arms	Sex and genotype, arm Fig. 3*M*	Three-way ANOVA; no main effect of sex: *F*_(1,131)_ = 0.00005, *p* = 0.994; no main effect of genotype: *F*_(2,131)_ = 0.009, *p* = 0.990; no main effect of arm: *F*_(1,131)_ = 628.018, *p* < 0.0001; no main effect of sex × genotype interaction: *F*_(2,131)_ = 0.005, *p* = 0.994; no main effect of sex × arm: *F*_(1,131)_ = 1.015, *p* = 0.315; no main effect of genotype × arm: *F*_(1,131)_ = 0.174, *p* = 0.840; no main effect of genotype × sex × arm: *F*_(2,131)_ = 1.549, *p* = 0.216
Shank3+ (*n* = 24 WT, 19 HET, 16 KO)		Duration in arms	Sex and genotype, arm Fig. 3*N*	Three-way ANOVA; no main effect of sex: *F*_(1,105)_ = 0.087, *p* = 0.768; no main effect of genotype: *F*_(2,105)_ = 0.314, *p* = 0.730; no main effect of arm: *F*_(1,105)_ = 708.548, *p* < 0.0001; no main effect of sex × genotype interaction: *F*_(2,105)_ = 0.358, *p* = 0.699; no main effect of sex × arm: *F*_(1,105)_ = 1.521, *p* = 0.220; no main effect of genotype × arm: *F*_(1,105)_ = 2.244, *p* = 0.111; no main effect of genotype × sex × arm: *F*_(2,105)_ = 1.482, *p* = 0.232
Shank3– (*n* = 24 WT,16 HET, 20 KO)	Rearing	Habituation of rearing	Sex, genotype, bin *Post hoc*: Scheffe Genotype Fig. 3*O*	Three-way ANOVA; **main effect of sex: *F*_(1,317)_ = 20.972, *p* < 6.99E-6; main effect of genotype: *F*_(2,317)_ = 11.877, *p* < 0.00001**; **main effect of bin: *F*_(5,317)_ = 13.643, *p* < 6.20E-12; main effect of sex × genotype interaction: *F*_(2,317)_ = 10.771, *p* < 0.00003**; no main effect of sex × bin: *F*_(5,317)_ = 0.472, *p* = 0.796; no main effect of genotype × bin: *F*_(10,317)_ = 0.142, *p* = 0.999; no main effect of genotype × sex × bin: *F*_(10,317)_ = 0.173, *p* = 0.997 **WT vs HET: *p* < 0.0004** **WT vs KO: *p* < 0.0002** HET vs KO: *p* = 0.997
Shank3+ (*n* = 24 WT, 19 HET, 20 KO)	Rearing	Habituation of rearing	Sex, genotype, bin Fig. 3*P*	Three-way ANOVA; no main effect of sex: *F*_(1,255)_ = 0.342, *p* = 0.322; no main effect of genotype: *F*_(2,255)_ = 0.781, *p* = 0.130; **main effect of bin: *F*_(23,255)_ = 78.67, *p* < 0.00001**; no main effect of sex × genotype interaction: *F*_(2,255)_ = 0.791, *p* = 0.242; **main effect of sex × bin: *F*_(23,255)_ = 1.39, *p* < 0.0031;** no main effect of genotype × bin:*F*_(46,255)_ = 1.13, *p* = 0.217; no main effect of genotype × sex ×bin: *F*_(46,255)_ = 1.223, *p* = 0.0691
Shank3– (*n* = 24 WT, 16 HET, 20 KO)	Rotarod	Latency to fall	Sex, genotype, trial Fig. 3*S*	Three-way ANOVA; **main effect of sex: *F*_(1,455)_ = 25.152, *p* < 7.91E-7**; no main effect of genotype: *F*_(2,455)_ = 0.912, *p* = 0.387; **main effect of trial: *F*_(1,455)_ = 4.26, *p* < 0.00015**; no main effect of sex × genotype interaction: *F*_(2,455)_ = 0.9259, *p* = 0.397; no main effect of sex × trial: *F*_(1,455)_ = 0.964, *p* = 0.456; no main effect of genotype × trial: *F*_(1,131)_ = 0.757, *p* = 0.7157; no main effect of genotype × sex × arm: *F*_(2,455)_ = 0.279, *p* = 0.9957
Shank3+ (*n* = 24 WT, 19 HET, 16 KO)	Rotarod	Latency to fall	Sex, genotype, trial Fig. 3*T*	Three-way ANOVA; **main effect of sex: *F*_(1,463)_ = 21.576, *p* < 4.567E-6**; no main effect of genotype: *F*_(2,463)_ = 2.338, *p* = 0.097; **main effect of trial: *F*_(7,463)_ = 14.727, *p* < 0.00001**; **main effect of sex × genotype interaction: *F*_(2,463)_ = 3.147, *p* < 0.043;** no main effect of sex × trial: *F*_(7,463)_ = 0.548, *p* = 0.797; no main effect of genotype × trial: *F*_(14,463)_ = 0.123, *p* = 0.999; no main effect of genotype × sex × trial: *F*_(14,463)_ = 0.397, *p* = 0.975
Shank3– (*n* = 24 WT, 16 HET, 20 KO)	Locomotor	Beam breaks	Sex, genotype, bin *Post hoc* Scheffe Genotype Sex Female Male Fig. 3*Q*	Three-way ANOVA; **main effect of sex: *F*_(1,1583)_ = 3.847, *p* < 0.00004; main effect of genotype: *F*_(2,1583)_ = 42.36, *p* < 0.00001; main effect of bin: *F*_(23,1583)_ = 57.45, *p* < 0.00001**; **main effect of sex × genotype interaction: *F*_(2,1583)_ = 8.596, *p* < 0.0001;** no main effect of sex × bin: *F*_(23,1583)_ = 0.317, *p* = 0.999; no main effect of genotype × bin: *F*_(46,1583)_ = 0.915, *p* = 0.634; no main effect of genotype × sex × bin: *F*_(46,1583)_ = 0.528, *p* = 0.996 **HET vs KO: *p* < 0.0006** **HET vs WT: *p* < 0.00001** **KO vs WT: *p* < 0.00004** WT: M vs F: *p* = 0.880 HET: M vs F: *p* = 0.650 KO: M vs F: *p* = 0.194 HET vs KO: *p* = 0.968 HET vs WT: *p* = 0.591 KO vs WT: *p* = 0.419 HET vs KO: *p* = 0.848 HET vs WT: *p* = 0.891 KO vs WT: *p* = 0.987
Shank3+ (*n* = 24 WT, 19 HET, 20 KO)		Beam breaks	Sex, genotype, bin *Post hoc* Scheffe Genotype Sex male vs female Female Male Fig. 3*R*	Three-way ANOVA; no main effect of sex: *F*_(1,1271)_ = 2.396, *p* = 0.121; **main effect of genotype: *F*_(2,1271)_ = 3.489, *p* < 0.030**; **main effect of bin: *F*_(23,1271)_ = 105.61, *p* < 0.00001**; **main effect of sex × genotype interaction: *F*_(2,1271)_ = 49.89, *p* < 0.00001**; no main effect of sex × bin: *F*_(23,1271)_ = 2.39, *p* < 0.00025; no main effect of genotype × bin: *F*_(46,1271)_ = 1.145, *p* = 0.237; **main effect of genotype × sex × bin: *F*_(46,1271)_ = 5.793, *p* < 0.00001** HET vs KO: *p* = 0.989 HET vs WT: *p* = 0.119 KO vs WT: *p* < 0.053 WT: *p* = 0.737 HET: *p* = 0.598 KO: *p* = 0.286 HET vs KO: *p* = 0.525 HET vs WT: *p* = 0.833 KO vs WT: *p* = 0.801 HET vs KO: *p* = 0.896 HET vs WT: *p* = 0.949 KO vs WT: *p* = 0.716
Shank3– (*n* = 24 WT, 16 HET, 20 KO)	Morris water maze	Latency to reach platform	Sex, genotype, days *Post hoc*: Scheffe Genotype Sex: female vs male Female Male Fig. 4*A*	Three-way ANOVA; no main effect of sex: *F*_(1,543)_ = 0.994, *p* = 0.319; **main effect of genotype: *F*_(2,543)_ = 6.600, *p* < 0.0014**; **main effect of day: *F*_(8,543)_ = 33.361, *p* < 0.00001**; **main effect of sex × genotype interaction: *F*_(2,543)_ = 3.830, *p* < 0.022;** no main effect of sex × day: *F*_(8,543)_ = 1.012, *p* = 0.425; no main effect of genotype × day: *F*_(16,543)_ = 0.424, *p* = 0.976; no main effect of genotype × sex × day: *F*_(16,543)_ = 1.21, *p* = 0.254 HET vs KO: *p* = 0.088 HET vs WT: *P* = 0.471 **KO vs WT: *p* < 0.001** WT: *p* = 0.572 HET: *p* = 0.488 KO: *p* = 0.230 HET vs KO: *p* = 0.462 HET vs WT: *p* = 0.518 KO vs WT: *p* = 0.076 HET vs KO: *p* = 0.837 HET vs WT: *p* = 0.946 KO vs WT: *p* = 0.940
		% Thigmotaxis	Sex, genotype, days *Post hoc*: Scheffe Genotype Sex: female vs male Female Male Fig. 4*B*	Three-way ANOVA; **main effect of sex: *F*_(1,543)_ = 9.135, *p* < 0.0026**; **main effect of genotype: *F*_(2,543)_ = 13.132, *p* < 2.78E-6**; **main effect of day: *F*_(8,543)_ = 80.565, *p* < 0.00001**; no main effect of sex × genotype interaction: *F*_(2,543)_ = 2.089, *p* = 0.124; **main effect of sex × day: *F*_(8,543)_ = 2.738, *p* < 0.0057**; no main effect of genotype × day: *F*_(16,543)_ = 0.538, *p* = 0.926; no main effect of genotype × sex ×day: *F*_(16,543)_ = 0.866, *p* = 0.608 HET vs KO: *p* = 0.124 **HET vs WT: *p* < 0.018** **KO vs WT: *p* < 3.698E-6** **WT: *p* < 0.032** HET: *p* = 0.647 KO: *p* = 0.260 HET vs KO: *p* = 0.291 HET vs WT: *p* = 0.718 KO vs WT: *p* = 0.070 HET vs KO: *p* = 0.800 HET vs WT: *p* = 0.319 **KO vs WT: *p* < 0.016**
		Mean velocity	Sex, genotype, days *Post hoc*: Scheffe Genotype Sex: female vs male Female KO vs WT: *p* < 0.013 Fig. 4*C*	Three-way ANOVA; **main effect of sex: *F*_(1,543)_ = 4.993, *p* < 0.025**; **main effect of genotype: *F*_(2,543)_ = 5.019, *p* < 0.006**; **main effect of day: *F*_(8,543)_ = 2.425, *p* < 0.014**; no main effect of sex × genotype interaction: F(2,543) = 0.305, *p* = 0.737; no main effect of sex × day: *F*_(8,543)_ = 0.686, *p* = 0.703; no main effect of genotype × day: *F*_(16,543)_ = 1.411,*p* = 0.130; no main effect of genotype × sex × day: *F*_(16,543)_ = 0.878,*p* = 0.594 HET vs KO: *p* = 0.092 HET vs WT: *p* = 0.761 **KO vs WT: *p* < 0.008** **WT: *p* = 0.083** HET: *p* = 0.118 KO: *p* = 0.935 HET vs KO: *p* = 0.094 HET vs WT: *p* = 0.697 KO vs WT: *p* < 0.013 HET vs KO: *p* = 0.999 HET vs WT: *p* = 0.695 KO vs WT: *p* = 0.542
		Total distance	Sex, genotype, days *Post hoc*: Scheffe Genotype Sex: female vs male Female Male Fig. 4*D*	Three-way ANOVA; no main effect of sex: *F*_(1,543)_ = 0.954, *p* = 0.386; **main effect of genotype: *F*_(2,543)_ = 11.954, *p* < 8.528E-6**; **main effect of day: *F*_(8,543)_ = 34.530, *p* < 0.00001**; **main effect of sex × genotype interaction: *F*_(2,543)_ = 5.956, *p* < 0.0027**; no main effect of sex × day: *F*_(8,543)_ = 1.140, *p* = 0.334; no main effect of genotype × day: *F*_(16,543)_ = 1.003, *p* = 0.451; no main effect of genotype × sex × day: *F*_(16,543)_ = 1.321, *p* = 0.178 HET vs KO: *p* < 0.015 HET vs WT: *p* = 0.221 **KO vs WT: *p* < 8,712E-6** **WT: *p* = 0.394** HET: *p* = 0.541 KO: *p* = 0.503 HET vs KO: *p* = 0.951 HET vs WT: *p* = 0.222 KO vs WT: *p* = 0.181 HET vs KO: *p* = 0.962 HET vs WT: *p* = 0.996 KO vs WT: *p* = 0.968
		Probe trial	Sex, genotype, location *Post hoc*: Scheffe Location Fig. 4*E*	Three-way ANOVA; no main effect of sex: *F*_(1,183)_ = 0.093, *p* = 0.760; **main effect of genotype: *F*_(2,183)_ = 1.446, *p* = 0.238**; **main effect of location: *F*_(3,183)_ = 276.264, *p* < 0.00001**; no main effect of sex × genotype interaction: *F*_(2,183)_ = 1.228, *p* = 0.295; no main effect of sex × location: *F*_(3,183)_ = 1.683, *p* = 0.172; no main effect of genotype × location: *F*_(6,183)_ = 0.729, *p* = 0.626; no main effect of genotype × sex × location: *F*_(6,183)_ = 1.159, *p* = 0.330 **Far vs near: *p* < 0.00001** **Far vs opp: *p* < 0.00001** **Far vs Target: *p* < 0.00001** Near vs opp: *p* = 0.915 **Near vs Target: *p* < 0.00001** **Opp vs Target: *p* < 0.00001**
Shank3+ (*n* = 24 WT, 19 HET, 20 KO)	Morris water maze	Latency to reach platform	Sex, genotype, days *Post hoc*: Scheffe Genotype Fig. 4*F*	Three-way ANOVA; no main effect of sex: *F*_(1,315)_ = 1.136, *p* = 0.287; **main effect of genotype: *F*_(2,315)_ = 4.048, *p* < 0.018**; **main effect of day: *F*_(6,315)_ = 13.185, *p* < 0.00001**; no main effect of sex × genotype interaction: *F*_(2,315)_ = 0.383, *p* = 0.961; no main effect of sex × day: *F*_(6,315)_ = 1.500, *p* = 0.177; no main effect of genotype × day: *F*_(12,315)_ = 0.709, *p* = 0.742; no main effect of genotype × sex × day: *F*_(12,315)_ = 0.772, *p* = 0.678 **HET vs KO: *p* < 0.018** HET vs WT: *p* = 0.289 KO vs WT: *p* = 0.372
		% Thigmotaxis	Sex, genotype, days *Post hoc*: Scheffe Genotype Sex: female vs male Female Male Fig. 4*G*	Three-way ANOVA; **main effect of sex: *F*_(1,315)_ = 8.982, *p* < 0.0029; main effect of genotype: *F*_(2,315)_ = 5.746, *p* < 0.0035**; **main effect of day: *F*_(6,315)_ = 51.074, *p* < 0.00001**; **main effect of sex × genotype interaction: *F*_(2,315)_ = 3.707, *p* < 0.025**; no main effect of sex × day: *F*_(6,315)_ = 1.657, *p* = 0.131; no main effect of genotype × day: *F*_(12,315)_ = 0.617, *p* = 0.827; no main effect of genotype × sex × day: *F*_(12,315)_ = 0.440, *p* = 0.946 **HET vs KO: *p* < 0.004** HET vs WT: *p* = 0.530 KO vs WT: *p* = 0.064 **WT: *p* < 0.025** HET: *p* = 0.587 KO: *p* = 0.221 HET vs KO: *p* = 0.374 HET vs WT: *p* = 0.945 KO vs WT: *p* = 0.588 HET vs KO: *p* = 0.085 HET vs WT: *p* = 0.102 KO vs WT: *p* = 0.969
		Mean velocity	Sex, genotype, days Fig. 4*H*	Three-way ANOVA; no main effect of sex: F(1,315) = 0.219, *p* = 0.640; **main effect of genotype: *F*_(2,315)_ = 0.857, *p* = 0.857**; **main effect of day: *F*_(8,315)_ = 0.940, *p* = 0.466**; no main effect of sex × genotype interaction: *F*_(2,315)_ = 0.438, *p* = 0.645; no main effect of sex × day: *F*_(6,543)_ = 0.763, *p* = 0.763; no main effect of genotype × day: *F*_(12,315)_ = 0.922, *p* = 0.524; no main effect of genotype × sex × day: *F*_(12,315)_ = 1.014, *p* = 0.435
		Total distance	Sex, genotype, days *Post hoc*: Scheffe Genotype Fig. 4*I*	Three-way ANOVA; no main effect of sex: *F*_(1,315)_ = 2.447, *p* = 0.118; **main effect of genotype: *F*_(2,315)_ = 3.673, *p* < 0.026**; **main effect of day: *F*_(6,315)_ = 10.167, *p* < 3.717E-10**; no main effect of sex × genotype interaction: *F*_(2,315)_ = 0.435, *p* = 0.647; no main effect of sex × day: *F*_(6,315)_ = 1.527, *p* = 0.169; no main effect of genotype × day: *F*_(12,315)_ = 0.853, *p* = 0.594; no main effect of genotype × sex × day: *F*_(12,315)_ = 0.870, *p* = 0.577 **HET vs KO: *p* < 0.028** HET vs WT: *p* = 0.227 KO vs WT: *p* = 0.550
		Probe trial	Sex, genotype, location *Post hoc*: Scheffe Location Fig. 4*J*	Three-way ANOVA; no main effect of sex: *F*_(1,183)_ = 2.158, *p* = 0.143; **main effect of genotype: *F*_(2,183)_ = 1.446, *p* = 0.238**; **main effect of zone: *F*_(3,183)_ = 276.175, *p* < 0.00001**; no main effect of sex × genotype interaction: *F*_(2,183)_ = 0.058, *p* = 0.942; no main effect of sex × zone: *F*_(3,183)_ = 1.680, *p* = 0.173; main effect of genotype × zone: *F*_(6,183)_ = 0.729, *p* = 0.626; no main effect of genotype × sex × zone: F(6, 183) = 1.196, *p* = 0.310 **Far vs near: *p* < 0.00001** **Far vs opp: *p* < 0.00001** **Far vs target: *p* < 0.00001** Near vs opp: *p* = 0.915 **Near vs target: *p* < 0.00001** **Opp vs target: *p* < 0.00001**
Shank3– (*n* = 24 WT, 16 HET, 20 KO)	Fear conditioning	Cue test	Genotypes and sex Fig. 5*A*	Two-way ANOVA; no main effect of sex: *F*_(1,52)_ = 2.774, *p* = 0.102; no main effect of genotype: *F*_(2,52)_ = 1.991, *p* = 0.147; no main effect of sex × genotype interaction: *F*_(1,52)_ = 0.669, *p* = 0.516
		Contextual test	Genotypes and sex Fig. 5*B*	Two-way ANOVA; no main effect of sex: *F*_(1,64)_ = 1.426, *p* = 0.237; no main effect of genotype: *F*_(2,64)_ = 0.423, *p* = 0.656; no main effect of sex × genotype interaction: *F*_(1,64)_ = 1.347, *p* = 0.2677
Shank3+ (*n* = 24 WT, 19 HET, 20 KO)	Fear conditioning	Cue test	Genotypes and sex Fig. 5*C*	Two-way ANOVA; no main effect of sex: *F*_(1,52)_ = 2.774, *p* = 0.102; no main effect of genotype: *F*_(2,52)_ = 1.991, *p* = 0.147; no main effect of sex × genotype interaction: *F*_(1,52)_ = 0.669, *p* = 0.516
		Contextual test	Genotypes and sex Fig. 5*D*	Two-way ANOVA; no main effect of sex: *F*_(1,52)_ = 0.0007, *p* = 0.978; no main effect of genotype: *F*_(2,52)_ = 1.586, *p* = 0.215; no main effect of sex × genotype interaction: *F*_(1,52)_ = 2.491, *p* = 0.0936
Shank3– (*n* = 24 WT, 16 HET, 20 KO)	Nesting	Width	Genotypes, sex, time *Post hoc*: Scheffe Genotype Time Fig. 5*F*	Three-way ANOVA; **main effect of sex: *F*_(1,197)_ = 6.559, *p* < 0.011; main effect of genotype: *F*_(2,197)_ = 3.076, *p* < 0.048**; **main effect of time: *F*_(2,197)_ = 17.297, *p* < 1.344E-7**; no main effect of sex × genotype interaction: *F*_(2,197)_ = 0.768, *p* = 0.465; no main effect of sex × time: *F*_(2,197)_ = 0.461, *p* = 0.631; no main effect of genotype × time: *F*_(4,197)_ = 0.086, *p* = 0.986; no main effect of genotype × sex × time: *F*_(4,197)_ = 0.202, *p* = 0.936 HET vs KO: *p* = 0.888 HET vs WT: *p* = 0.212 KO vs WT: *p* = 0.070 **30 vs 60: *p* < 0.003** **30 vs 90: *p* < 1.357E-7** **60 vs 90: *p* < 0.047**
		Height	Genotypes, sex, time *Post hoc*: Scheffe Genotype Fig. 5*E*	Three-way ANOVA; no main effect of sex: *F*_(1,197)_ = 0.448, *p* = 0.504; **main effect of genotype: *F*_(2,197)_ = 3.952, *p* < 0.020**; **main effect of time: *F*_(2,197)_ = 4.844, *p* < 0.008**; no main effect of sex × genotype interaction: *F*_(2,197)_ = 0.241, *p* = 0.785; no main effect of sex × time: *F*_(2,197)_ = 0.342, *p* = 0.710; no main effect of genotype × time: *F*_(4,197)_ = 0.140, *p* = 0.966; no main effect of genotype × sex × time: *F*_(4,197)_ = 0.324, *p* = 0.861 30 vs 60: *p* = 0.959 **30 vs 90: *p* < 0.046** 60 vs 90: *p* = 0.087
Shank3+ (*n* = 24 WT, 19 HET, 20 KO)	Nesting	Width	Genotypes, sex, time *Post hoc*: Scheffe Time Fig. 5*H*	Three-way ANOVA; **main effect of sex: *F*_(1,152)_ = 8.139, *p* < 0.005**; **main effect of genotype: *F*_(2,152)_ = 1.237, *p* = 0.293**; **main effect of time: *F*_(2,152)_ = 15.108, *p* < 1.199E-6**; no main effect of sex × genotype interaction: *F*_(2,152)_ = 2.140, *p* = 0.093; no main effect of sex × time: *F*_(2,152)_ = 0.287, *p* = 0.750; no main effect of genotype × time: *F*_(4,152)_ = 0.166, *p* = 0.955; no main effect of genotype × sex × time: *F*_(4,152)_ = 0.540, *p* = 0.706 **30 vs 60: *p* < 0.0086** **30 vs 90: *p* < 1.146E-6** 60 vs 90: *p* = 0.060
		Height	Genotypes and sex *Post hoc*: Scheffe Time Fig. 5*G*	Three-way ANOVA; **main effect of sex: *F*_(1,152)_ = 9.234, *p* < 0.0028; main effect of genotype: *F*_(2,152)_ = 0.117, *p* = 0.889**; **main effect of time: *F*_(2,152)_ = 22.485, *p* < 3.7277E-9; main effect of sex × genotype interaction: *F*_(2,152)_ = 5.748, *p* < 0.0040**; no main effect of sex × time: *F*_(2,152)_ = 0.479, *p* = 0.750; no main effect of genotype × time: *F*_(4,152)_ = 0.286, *p* = 0.886; no main effect of genotype × sex × time: *F*_(4,152)_ = 1.582, *p* = 0.122 **30 vs 60: *p* < 0.008** **30 vs 90: *p* < 0.017** 60 vs 90: *p* = 0.762
Shank3– (*n* = 24 WT, 16 HET, 20 KO)	Cookie finding test	Latency to find the cookie	Genotypes and sex Fig. 5*I*	Two-way ANOVA; no main effect of sex: *F*_(1,60)_ = 0.526, *p* = 0.471; no main effect of genotype: *F*_(2,60)_ = 0.301, *p* = 0.741; no main effect of sex × genotype interaction: *F*_(1,60)_ = 0.621, *p* = 0.540
Shank3+ (*n* = 24 WT, 19 HET, 20 KO)		Latency to find the cookie	Genotypes and sex Fig. 5*J*	Two-way ANOVA; no main effect of sex: *F*_(1,64)_ = 0.689, *p* = 0.409; no main effect of genotype: *F*_(2,64)_ = 0.640, *p* = 0.530; no main effect of sex × genotype interaction: *F*_(1,64)_ = 0.004, *p* = 0.995
Shank3– (*n* = 24 WT, 16 HET, 20 KO)	Marble burying	Marbles buried	Genotypes and sex Fig. 5*K*	Two-way ANOVA; no main effect of sex: *F*_(1,65)_ = 0.029, *p* = 0.864; no main effect of genotype: *F*_(2,65)_ = 0.843, *p* = 0.435; no main effect of sex × genotype interaction: *F*_(1,65)_ = 0.214, *p* = 0.807
Shank3+ (*n* = 24 WT, 19 HET, 16 KO)	Marble burying	Marbles buried	Genotypes and sex Fig. 5*L*	Two-way ANOVA; no main effect of sex: *F*_(1,60)_ = 0.229, *p* = 0.633; no main effect of genotype: *F*_(2,60)_ = 1.149, *p* = 0.324; no main effect of sex × genotype interaction: *F*_(1,60)_ = 0.616, *p* = 0.543

### Behavioral tests

#### Elevated plus maze

This test was conducted as described previously (Etherton et al., 2009). Briefly, mice were placed in the center of the maze (each arm was 30 cm long and 5 cm wide with 25-cm-high walls on the closed arms) and allowed to freely explore for 5 min. All mice were tested under dim white light at ∼7 lux. Noldus Ethovision version 3.1 was used to track and record mouse behavior.

#### Locomotor and rearing activity

Locomotor activity was measured as described previously (Powell et al., 2004; Tabuchi et al., 2007; Etherton et al., 2009). Mice were placed in novel cages (a clean cage with the same dimensions as their home cage; L × W × H = 27.3 × 16.5 × 12.7 cm) with minimal bedding and allowed to freely explore for 2 h under red lighting. Horizontal locomotor activity (i.e., the number of photobeam breaks) was measured by computer software (San Diego Instruments) and data were analyzed in 5-min bins.

#### Dark/light test

The dark/light test was conducted as described previously (Powell et al., 2004; Blundell et al., 2009). Mice were placed in the dark chamber (each chamber was 25 × 26 cm with 2066 lux on the light side and ∼1 lux on the dark side) and allowed to habituate for 2 min. After habituation, mice were allowed to freely explore both chambers for 10 min.

#### Rotarod

The rotarod test was conducted as described previously (Powell et al., 2004). Briefly, mice were placed on a stationary rotarod (IITC Life Science) that was then activated and accelerated from 0 to 45 rpm over 5 min. The latency for the mice to fall off the rod was measured. If a mouse held onto the rotating rod for one complete revolution it was scored as a fall. Each mouse received four trials per day for 2 d. Within a day, each trial had an intertrial interval of ∼45 min.

#### OF

The OF test was conducted as described previously (Etherton et al., 2009). Briefly, mice were placed along the edge of an open arena (44 × 44 × 44 cm, ∼7 lux) and allowed to freely explore for 10 min. Mice were monitored using CleverSys TopScan Software.

#### Social interaction tests

Social interaction with a novel juvenile target mouse was performed essentially as described (Kwon et al., 2006; Tabuchi et al., 2007; Blundell et al., 2009). Briefly, following a 15-min habituation under red light, the experimental and target mice (novel BALB/cByJ juvenile mouse; three weeks of age; The Jackson Laboratory, stock #001026) were placed in a novel cage for 2 min and allowed to directly interact. Interaction was scored by observing the duration and number of times the test mouse-initiated contact with or sniffed the juvenile mouse. Contact was considered as any part of the body touching the juvenile mouse. Three days later, the same experimental and juvenile mice were paired again in a novel cage for 2 min and scored in the same manner. Social interaction with a caged adult was performed as described (Blundell et al., 2010). Briefly, the test was performed in a 48 × 48 cm^2^ white plastic arena under red light using 3.5” × 2” × 30” clear rectangular cage containing a BALB/cByJ novel adult mouse. The lower half of the rectangular cage has small openings to allow for olfactory and minimal tactile interaction. Initially mice were allowed to explore the arena for 5 min with an empty rectangular cage. Then mice were allowed to approach a novel adult mouse housed in the rectangular cage for another 5 min. Social interaction with genotype-matched and sex-matched pairs was performed by pairing mice with a sex-matched and genotype-matched partner within the experimental cohort. Matched pairs were derived from separate cages and were never previously housed together. Mouse pairs were placed at separate ends in an OF arena (44 × 44 × 44 cm) and allowed to interact for 5 min under dim lighting (∼7 lux). For three-chamber social approach, social preference and social novelty were tested using a three-chambered box as described previously (Blundell et al., 2009) and based to a large extent on the original descriptions (Moy et al., 2004; Nadler et al., 2004). This test consisted of three, 10-min trials. During the first trial, the mouse was allowed to explore the three-chamber box in which each end-chamber contained an empty cage (upside down pencil holder). In the second trial, the three-chamber box contained a novel BALB/cByJ stimulus mouse under a cage in one of the end-chambers and an empty cage in the opposite end-chamber. The test mouse was free to choose between an inanimate cage and a caged social target. For the third trial, the test mouse was free to choose between a caged novel social target (novel BALB/cByJ mouse) versus the same caged mouse in trial 2 (familiar social target). Locations of empty cages and social targets were counterbalanced, and mice were placed back into the home cage for very brief intervals between trials. Social interactions were objectively monitored and scored using video-tracking and automated CleverSys SocialScan Software.

#### Grooming

Mice were placed into an empty cage and allowed to habituate for 10 min in a room with ∼40 lux of white light. At the end of 10 min, the mice were monitored and video recorded for 10 min for later analysis. The number of grooming bouts along with the total time spent grooming was measured. Time spent grooming the face, head, body, or tail are all considered grooming.

#### Morris water maze

The Morris water maze task was conducted as previously described (Powell et al., 2004). Briefly, in a 120-cm diameter pool, a 10-cm diameter circular platform was submerged ∼1 cm below the surface of the water (22 ± 1°C) made opaque with white, non-toxic tempera paint. After finding the platform or being guided by the experimenter to the platform if the 60-s trial limit elapsed, mice remained on the platform for 15 s before being removed and returned to their home cage. Training was conducted over nine consecutive days, followed by a probe trail on day 10 (60-s swim with no platform). To test basic visual function, we measured the latency to reach the platform with a visible cue atop the platform in the water maze on day 10.

#### Marble burying

As described previously (Blundell et al., 2010b), individual mice were placed in a novel home cage with 5 cm of bedding. 20 black marbles (16 mm in diameter) were evenly placed on top of the bedding throughout the home cage, and mice were free to explore the cage for 30 min. After 30 min, the number of marbles buried was recorded. A marble was defined as buried when <25% of the marble was visible. This test was conducted in a dimly lit room (∼80 lux).

#### Fear conditioning

Fear conditioning was conducted as previously described (Powell et al., 2004). Mice were placed into a shock box with clear front and rear walls (medAssociates). After a 2-min exploration period, three 30-s, 90-dB acoustic conditioned stimuli (white noise) followed by a 2-s, 0.5-mA foot shock with 2-min interstimulus intervals were delivered. Mice remained in the chamber 2 min after the last conditioned stimulus/foot shock pairing. Freezing behavior (motionless except respirations) was monitored at 5-s intervals by an observer blind to the genotype. To test 24-h contextual memory, mice were placed into the same training box for 5 min and scored for freezing behavior every 5 s. Four hours following contextual memory testing, cue-dependent fear conditioning was tested. Mice were placed in a novel environment supplemented with vanilla odor for a 3-min baseline followed by 3 min of conditioned stimulus (tone). Freezing behavior was scored as contextual fear conditioning.

#### Nesting behavior

Mice were placed into a novel empty cage with a 5 × 5 cm square of pressed cotton (Nestlet; Ancare). The net increase in nest width and height were measured after 30, 60, and 90 min.

#### Olfactory cookie test

Mice were not habituated to the cookie before testing. Half of a cookie (Nutter Butter, Nabisco) was buried ∼1 cm under mouse bedding in a novel, clean mouse cage. A test mouse was placed in the cage, and the latency to find the cookie was recorded.

### Statistics

Statistical analyses of behavioral data were conducted using StatPlus software (version 6, AnalystSoft) using either two-way ANOVAs or three-way repeated measure, where applicable. *Post hoc* Scheffe contrast of means was applied for significant effects and interactions. Figures are represented as mean ± SEM.

## Results

### Generation of *Shank3* mutant mice

In our previous study, we successfully targeted disruption of the *Shank3* gene in mice by insertion of a transcriptional neo-stop cassette containing flanking loxP sites into intron 12, which we term Shank3^E13^ (Fig. 1*A*). We then created two separate lines of mice by crossing mice heterozygous for Shank3^E13^ with a NSE-tTA transgene mouse line (gift from Eric Nestler) or Tg(tetO-Cre)1Jaw/J transgene mouse line (The Jackson Laboratory, stock #006224), to generate Shank3^E13^+/−^,NSE-tTA+^ and Shank3^E13^+/−^,tetO+^, respectively. These mice were then bred together to generate mice positive for both transgenes, in addition to being WT (Shank3^E13^−/−^,^ *^tg^* ^++^; WT+), heterozygous (Shank3^E13^+/−^,^ *^tg^* ^++^; HET+), or homozygous (Shank3^E13+/+,^ *^tg^* ^++;^ KO+), for the floxed neo-stop cassette upstream of exon 13 of *Shank3*. The presence of both transgenes allowed for a 2-transgene regulatory system that can remove the floxed neo-stop cassette and thus restore the WT *Shank3* gene. In this 2-transgene regulatory system, NSE-tTA activation allows for the expression of tTA (tetracycline transactivator), which binds to the tetO (tetracycline-responsive promoter element) promoter that activates expression of cre-recombinase. Cre will bind to the loxP sites (Fig. 1, black triangles) flanking the neo-stop cassette and allow for removal of the cassette following recombination (Fig. 1*B*). Whole-lysates from the hippocampus, striatum, and cortex showed that control mice lacking one of the two transgenes (HET– and KO–) displayed reduced SHANK3 expression in all three brain regions. Mice containing both transgenes (WT+, HET+, and KO+), however, all displayed similar levels of SHANK3 expression compared with WT– mice (Fig. 1*C*, dashed line), thereby demonstrating complete rescue at the level of protein expression.

### Lack of brain region selectivity and expected doxycycline regulation

We initially chose this combination of NSE-tTA and TetO-cre transgenes for dual control purposes. First, based on previous studies (Chen et al., 1998), we expected this NSE-tTA transgene to express tTA largely limited to the striatum and cerebellum. Unfortunately, our data revealed a much more widespread NSE-tTA expression based on rescue of our Shank3^E13^ mutant (Fig. 1). Second, based on many published reports including our own (Monteggia et al., 2004), we anticipated that breeding and rearing mice on doxycycline-containing water would successfully suppress tTA activation of cre-recombinase expression so that we might use doxycycline withdrawal to regulate the temporal onset of genetic reversal by cre-recombinase. Our data using doxycycline revealed that the cre-recombinase expression was “leaky” and allowed for genetic reversal of Shank3^E13^ to WT *Shank3* even when doxycycline was provided (data not shown). Thus, we were only able to examine early developmental genetic rescue using this strategy.

### Social interaction tests in *Shank3* mutant mice

**Figure 2.**
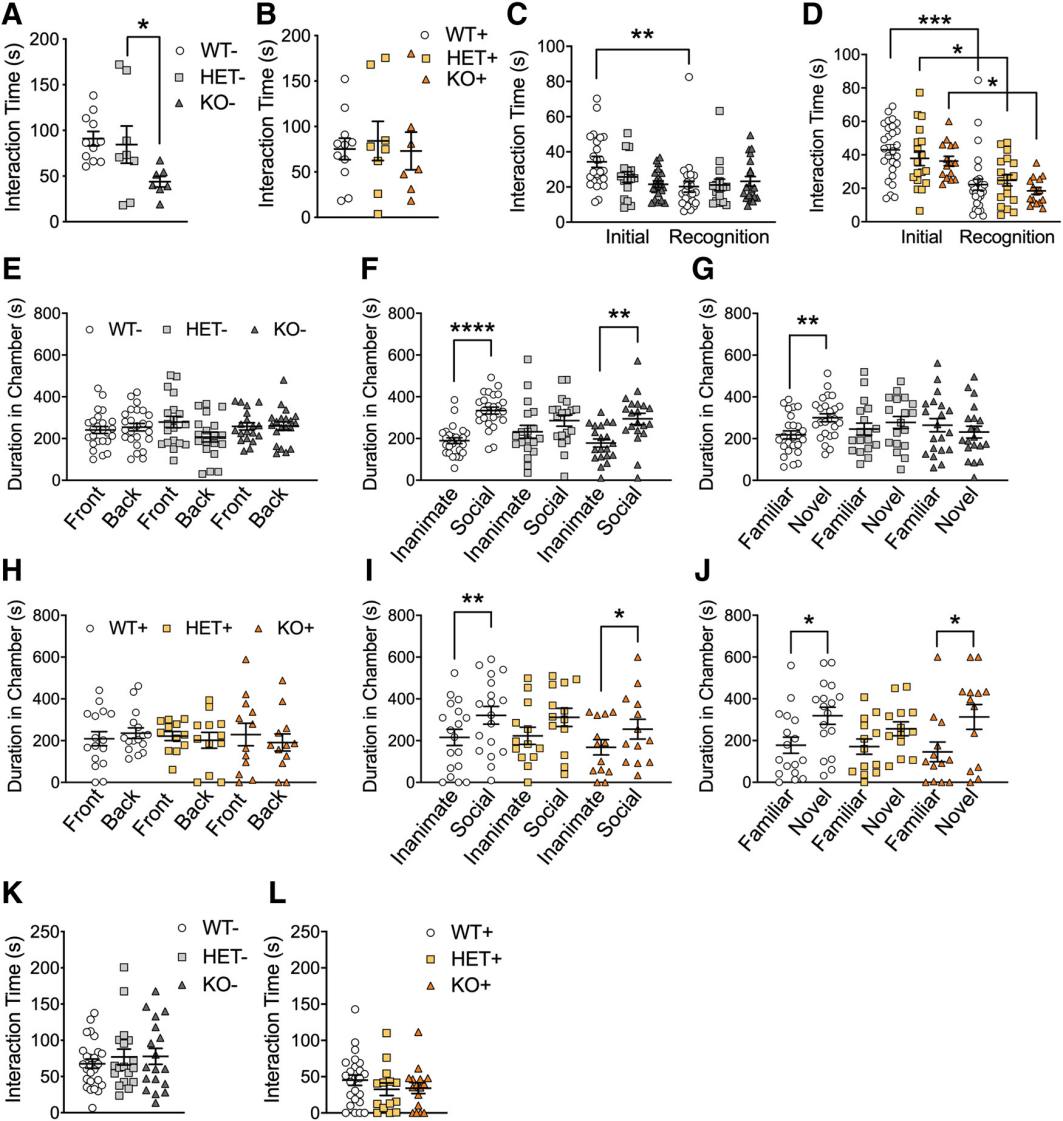
Rescue of social interaction deficits in *Shank3*+ mutant mice. In the genotype/sex-matched social interaction test, *Shank3* KO– mice displayed a significant reduction in social interaction time (***A***) compared with HET– mice. *Shank3*+ mice, including both genetic reversal transgenes, all displayed similar durations of social interaction (***B***) in the genotype/sex-matched social interaction test. In the social interaction with juvenile test of social memory, both HET and KO mice display deficits in social recognition (***C***). In the genetically reversed *Shank3*+ mice, however, all genotypes displayed similar levels of social memory (***D***). In the three-chamber test of sociability, *Shank3*– mice did not display a chamber preference in the initial trial (***E***). In second trial of this task, *Shank3* HET– mice did not display a preference for the social target over the inanimate object (***F***). In the third trial of this task, neither *Shank3* HET– nor KO– mice displayed a preference for social novelty (***G***). *Shank3*+ mice also did not display a baseline chamber preference in the initial trial of the three-chamber test (***H***). In the second trial, *Shank3* HET+ mice did not display a preference for the social target over the inanimate object (***I***). In the third trial of this task, *Shank3* HET+ mice did not display a preference for social novelty (***J***). *Shank3*– mice (***K***) along with *Shank3*+ mice (***L***) both display similar social interaction times, respectively, in approach of a novel, caged social target in the caged conspecific test. Data represented as mean ± SEM; *p < 0.05; **p < 0.01, ***p < 0.001, ****p < 0.0001; genotype sex match test *n* = 11 WT–, *n* = 8 HET–, *n* = 7 KO–; *n* = 11 WT+, *n* = 8 HET+, *n* = 7 KO+; all other behaviors *n* = 24 WT–, *n* = 16 HET–, *n* = 20 KO–, *n* = 24 WT+, *n* = 19 HET+, *n* = 16 KO+.

Considering that social deficits are a core feature of autism and a prominent feature of PMS, we tested *Shank3* mutant mice in social tasks. We assessed reciprocal social interaction by pairing mice of the same genotype and sex with one another for an initial encounter. *Shank3* KO– mice displayed a significant decrease in social interaction compared with HET– mouse pairs (total interaction: WT– vs KO–: *p* = 0.0508; HET– vs KO–: *p* = 0.012; WT– vs HET–: *p* = 0.750; Fig. 2*A*) a result that replicates our previous findings in Shank3^E13^KO mice. We did not find a significant difference in social interaction between WT– and KO– mice compared with our previous study (Jaramillo et al., 2017), however, there was a trend toward significance (*p* = 0.0508) between WT– and KO– mice. In our genetically reversed *Shank3* cohort (WT+, HET+, KO+), all three groups of mice displayed similar interaction times (Fig. 2*B*). These data indicate decreased reciprocal social interaction in Shank3 KO mice compared with Shank3 HET mice that was rescued with genetic reversal.

We next tested mutant mice in a social recognition memory task in which novel juvenile mice (three weeks of age) were introduced to experimental mice, then 3 d later the same juvenile mice were reintroduced to the same experimental mouse. *Shank3* WT– mice displayed a significant decrease in interaction time when the juvenile mouse was reintroduced, suggesting WT– mice displayed social recognition memory. Similar to our previously published findings, both HET– and KO– mice displayed a lack of social recognition memory (initial vs recognition time, WT–: *p* = 0.002, HET–: *p* = 0.317, KO–: *p* = 0.373; Fig. 2*C*). In our genetically reversed *Shank3* mice WT+, HET+, and KO+ mice all displayed significant levels of social recognition memory between the initial and recognition trials (initial vs recognition time, WT+: *p* = 0.024, HET+: *p* = 0.04, KO+: *p* = 0.019; Fig. 2*D*), suggesting effective genetic rescue of social recognition memory.

In our previous study, Shank^E13^ HET and KO mutant mice displayed abnormal social novelty exploration with HET mice also showing abnormalities in social versus inanimate preference in the three-chamber test of sociability (also known as three-box social interaction test; Jaramillo et al., 2017). We replicated these findings in the present study comparing Shank3 HET– and KO– mutant mice to WT– littermates (mice lacking at least one of the two transgenes needed for genetic rescue). In the initial trial to detect any baseline chamber bias, none of the groups displayed a bias (Fig. 2*E*). However, when one chamber contained a novel caged adult mouse and the other chamber contained an inanimate object, only the WT– and KO– mice displayed a preference for the social target. Replicating our previously published results (Jaramillo et al., 2017), the *Shank3* HET– mice did not display significant sociability or preference for the social target (social vs inanimate, WT: *p* < 0.00,001; HET–: *p* = 0.196, KO–: *p* < 0.0012; Fig. 2*F*). Additionally, when one of the chambers contained a familiar caged adult mouse, and the other chamber contained a novel caged adult mouse, only WT mice preferred the novel social target. *Shank3* HET– and KO– mice did not display a preference for social novelty (novel vs familiar WT–: *p* < 0.014, HET–: *p* = –0.703, KO–: *p* = 0.181; Fig. 2*G*).

In our genetically reversed *Shank3+* mice (containing both transgenes required for genetic rescue), all three groups of mice (WT+, HET+, KO+) displayed no initial chamber bias in the initial trial of the three-chamber test (Fig. 2*H*). In the social versus inanimate preference trial, both WT+ and KO+ mice displayed a preference for the social target, however, genetically rescued Shank3 HET+ mice still did not display a social preference (social vs inanimate WT+: *p* < 0.0044; HET+: *p* = 0.153, KO+: *p* = 0.042; Fig. 2*I*). In the social novelty trial, WT+ and KO+ mice preferred the novel social target. Although there was a trend toward preference for the novel target in HET+ mice, it was not significant. Thus, HET+ mice did not display a preference for social novelty (novel vs familiar WT+: *p* = 0.017, HET+: *p* = 0.099, KO+: *p* = 0.037; Fig. 2*J*). These findings replicate a lack of sociability in Shank3 HET mice and indicate that this particular deficit unique to heterozygotes was not reversible by genetic rescue of SHANK3 expression. Lastly, using a different social approach task referred to as “caged conspecific,” time spent approaching a novel mouse in an OF did not differ among genotypes (Fig. 2*K*,*L*), representing a failure to replicate the difference previously observed in homozygous Shank3^E13^ mice (Jaramillo et al., 2017).

### Restricted repetitive behaviors in *Shank3* mutant mice

**Figure 3.**
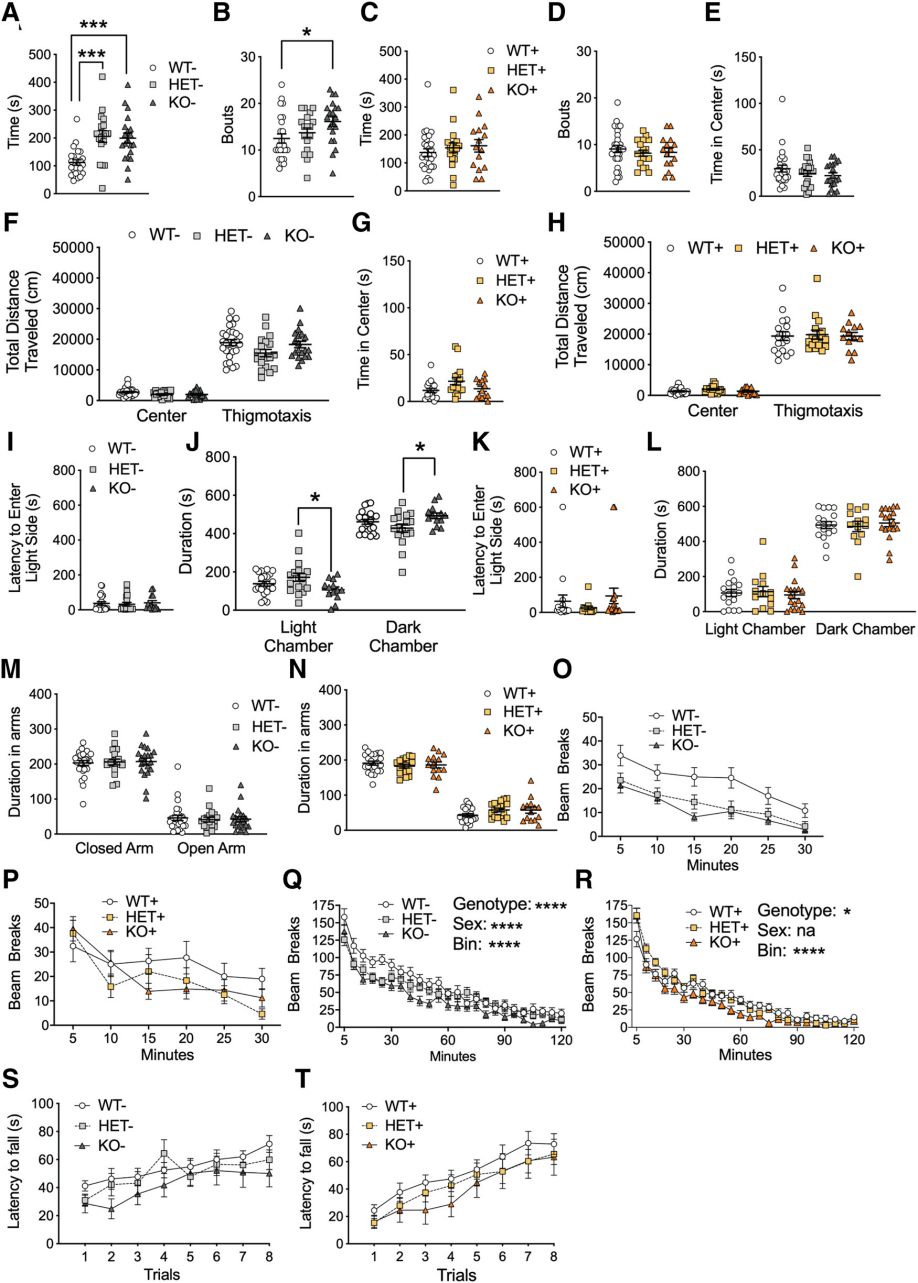
Rescue of increase repetitive grooming in *Shank3*+ mutant mice. ***A***, *Shank3* HET– and KO– mice displayed a significant increase in time spent grooming compared with WT– littermate mice. ***B***, KO– mice also display a significant increase in the number of grooming bouts compared with WT mice. ***C***, Genetically rescued *Shank3* WT+, HET+, and KO+ mice all displayed similar grooming duration and (***D***) number of grooming bouts. In the OF test, *Shank3*– mice all displayed similar duration in the center (***E***). Additionally, *Shank3*– mice displayed similar distances traveled in the center and thigmotaxis regions, respectively (***F***). *Shank3*+ mice displayed similar duration in the center of the OF (***G***) and similar distances traveled in the center and thigmotaxis regions (***H***). In the light dark test, *Shank3*– mice displayed similar latencies to enter the light side (***I***). *Shank3* HET– mice displayed increase duration in the light chamber compared with KO– mice (***J***). Additionally, HET– mice displayed a decrease in time spent in the dark chamber compared with KO– mice (***J***). None of the *Shank3*+ mice displayed a significant difference in their latency to enter the light side (***K***). Also, *Shank3*+ mice displayed similar duration in light chamber and dark chamber, respectively (***L***). In the elevated plus maze *Shank3*– mice displayed similar durations in the closed arm and open arm, respectively (***M***). *Shank3*+ mice displayed similar duration in closed arms and open arms, respectively (***N***). In the rearing test *Shank3* HET– and KO– mice displayed reduced rearing compared with WT– mice (***O***). *Shank3* HET+ and KO+ mice displayed similar rearing behavior when compared with WT+ mice (***P***). In the locomotor test KO– mice displayed a significant reduction in beam breaks over a 2-h time period when compared with HET– and WT– mice (***Q***). *Shank3* WT+, HET+ and KO+ mice all displayed similar levels of beam breaks (***R***). Both *Shank3*– (***S***) and *Shank3*+ (***T***) mice displayed similar latencies to fall of the rotarod, respectively. Data represented as mean ± SEM; *p < 0.05; **p < 0.01, ***p < 0.001, ****p < 0.0001, *n* = 24 WT–, *n* = 16 HET–, *n* = 20 KO–, *n* = 24 WT+, *n* = 19 HET+, *n* = 16 KO+.

Restricted and repetitive behaviors are another core feature of autism and PMS; thus, we monitored repetitive grooming behavior in *Shank3* mutant mice over a 10 min period. In our previous study (Jaramillo et al., 2017), Shank^E13^ HET and KO mice displayed increase grooming. In this study, we replicated these findings in both the HET– and KO– mutant mice. Both genotypes displayed a significant increase in time spent grooming compared with WT– littermates (WT– vs HET–: *p* = 0.0004; WT– vs KO–: *p* = 0.0007; HET– vs KO–: *p* = 0.968; Fig. 3*A*). Additionally, the increase in grooming time was accompanied by an increased number of grooming bouts in KO– mice (WT– vs KO–: *p* = 0.030, WT– vs HET–: *p* = 0.66, HET– vs KO– *p* = 0.265; Fig. 3*B*). Genetically reversed *Shank3* mutant mice displayed similar grooming durations (Fig. 3*C*) and bouts (Fig. 3*D*) across all three genotypes, again suggesting complete rescue of the grooming phenotype.

### Anxiety-like behavior tests in *Shank3* mutant mice

Like our previous publication, we found no changes in anxiety-related behaviors in the mutant and no changes with genetic reversal as expected. We examined multiple tasks of relevance to anxiety. In the OF test, all three *Shank3*– genotypes (WT–, HET–, and KO–) spent similar amounts of time in the central, anxiogenic region (Fig. 3*E*). Additionally, there were similar distances traveled among the three groups in both the center and thigmotaxis region during the OF test (Fig. 3*F*). The genetically reversed mice also displayed similar durations in the center of the OF (Fig. 3*G*) and similar total distances traveled in both the center and thigmotaxis regions (Fig. 3*H*).

In the dark/light test, WT–, HET–, and KO– all displayed a similar latency to enter the anxiety-provoking, light chamber (Fig. 3*I*). However, KO– mice spent significantly less time in the light chamber when compared with HET– mice (KO– vs HET–: *p* = 0.032, WT– vs HET–: *p* = 0.300, WT– vs KO– *p* = 0.395) and significantly more time in the dark chamber when compared with HET– mice (KO– vs HET–: *p* = 0.032, WT– vs HET–: *p* = 0.300, WT– vs KO– *p* = 0.395; Fig. 3*J*). In the genetically reversed *Shank3* mice, WT+, HET+, and KO+ all displayed similar durations in latency to enter the light side (Fig. 3*K*) and duration in the light and dark chambers, respectively (Fig. 3*L*). In the elevated plus maze test, WT–, HET–, and KO– all displayed similar durations in the closed and open arms (Fig. 3*M*). The genetic reversal *Shank3* mice, WT+, HET+, and KO+, also displayed similar durations in the closed and open arms (Fig. 3*N*).

Rearing activity in mice is thought to be a form of exploratory activity that could be linked to anxiety. In our previous studies, Shank3^E13^ HET and KO mice displayed decreased rearing events compared with WT mice (Jaramillo et al., 2017). We replicated these findings in *Shank3* HET– and KO– mice. Both displayed a significant reduction in rearing events over a 30 min period compared with WT– mice (WT– vs HET–: *p* = 0.0004, WT– vs KO–: *p* = 0.0002, HET– vs KO–: *p* = 0.997; Fig. 3*O*). In the genetically reversed mice, WT+, HET+, and KO+ all displayed similar levels of rearing activity (Fig. 3*P*), again demonstrating successful genetic reversal of this phenotype.

In the locomotor test, *Shank3* HET– and KO– mice displayed a significant reduction in locomotor activity over a 2-h time span compared with WT– mice (WT– vs HET–: *p* > 0.00,001, WT– vs KO–: *p* > 0.00,004, HET– vs KO–: *p* > 0.0006; Fig. 3*Q*) consistent with our previous findings (Jaramillo et al., 2017). In the genetically reversed *Shank3* mice, a main effect of genotype was also observed, though HET+ mice did not show a reduction in locomotor activity compared with WT, suggesting possible reversal (Fig. 3*R*). Lastly, we tested for motor abnormalities using the rotarod test, and observed no significant difference in latency to fall among WT–, HET–, and KO– mice (Fig. 3*S*) or among WT+, HET+, and KO+ mice, respectively (Fig. 3*T*). Thus, we failed to replicate our original finding of incoordination on the rotarod in the Shank3^E13^ mice (Jaramillo et al., 2017).

### *Shank3* mice and spatial learning

**Figure 4.**
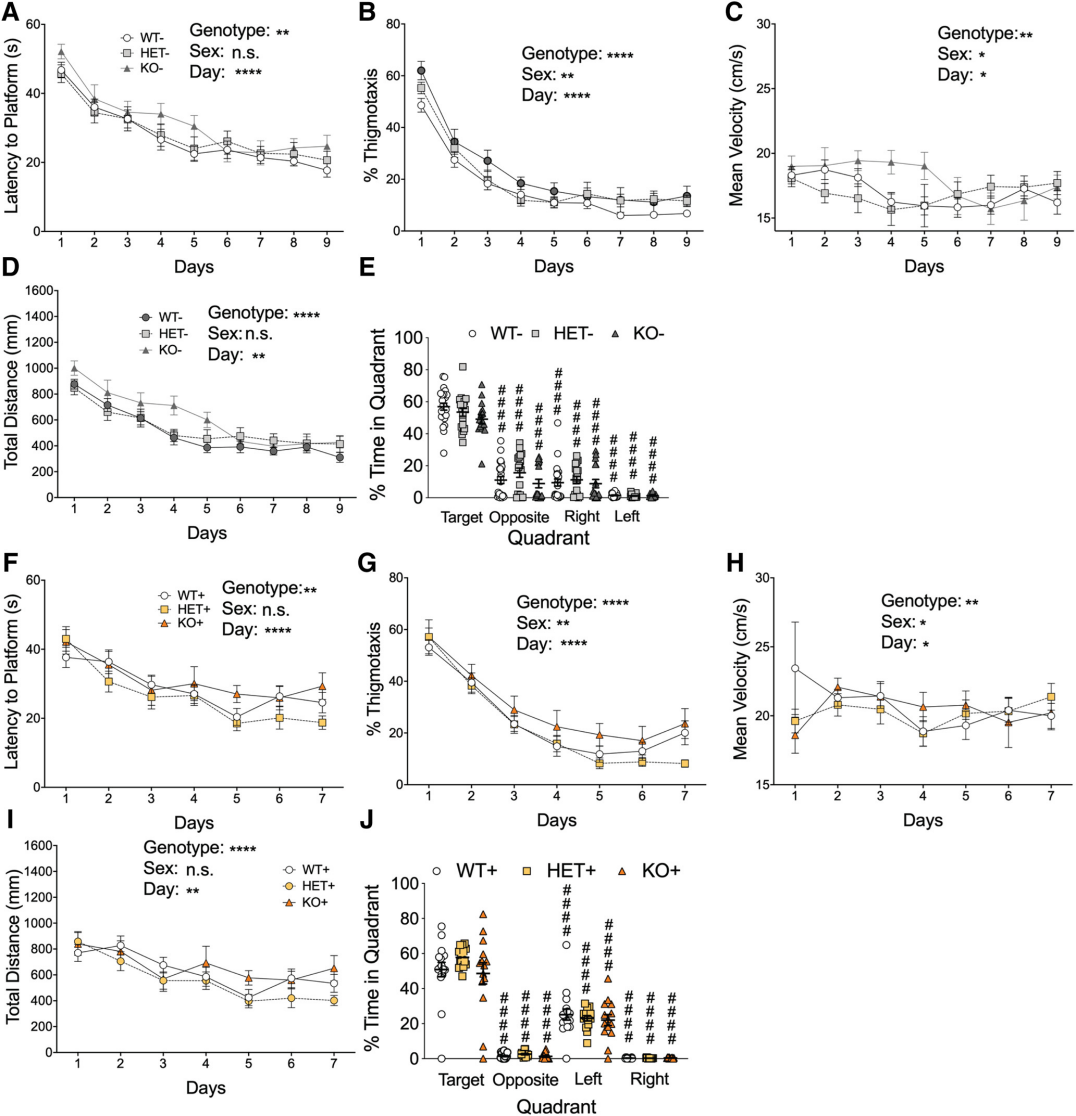
No alteration in spatial learning behavior in *Shank3*+ mutant mice. *Shank3*– mice all displayed similar levels of latency to reach the platform (***A***), percent time in the thigmotaxis region of the maze (***B***), mean velocity (***C***), and total distanced traveled (***D***) during the training phase of the Morris water maze test. In the probe trial, all three groups showed a preference for the target quadrant compared with other quadrants (***E***). *Shank3*+ mice all displayed similar levels of latency to reach the platform (***F***), percent time in the thigmotaxis region of the maze (***G***), mean velocity (***H***), and total distanced traveled (***I***) during the training phase of the Morris water maze test. ***J***, In the probe trial, all three groups showed preference for target quadrant. Data represented as mean ± SEM; ####*p* < 0.0001; *n* = 24 WT–, *n* = 16 HET–, *n* = 20 KO–, *n* = 24 WT+, *n* = 19 HET+, *n* = 16 KO+.

Recent studies by the CDC suggest ∼38% of children with ASD have intellectual disability (Christensen et al., 2018), the prevalence of which is even higher in PMS patients (Phelan and Rogers, 1993). Thus, we tested spatial learning and memory ability in the *Shank3* mutants using the Morris water maze. Unlike our previously published data, we did not find any major difference in spatial learning/memory in *Shank3* mutant mice. Although some main effects of genotype were observed statistically, the valence of such effects were not readily ascertained from the data. All mice in the control group showed similar latencies to reach the platform during the training phase of the test (Fig. 4*A*). Additionally, control mice showed similar percent time in the thigmotaxis zone (Fig. 4*B*), similar mean velocities (Fig. 4*C*), and similar distances traveled (Fig. 4*D*). In the probe trial, all three control genotypes displayed similar preference for the target quadrant versus all other quadrants (Fig. 4*E*). Genetically reversed *Shank3* mice also displayed similar times in latencies to the platform (Fig. 4*F*), percentage of time in the thigmotaxis zone (Fig. 4*G*), mean velocities (Fig. 4*H*), and total distances traveled (Fig. 4*I*). In the probe trial all three genotypes displayed a preference for the target quadrant compared with all other quadrants (Fig. 4*J*). This represents a failure to replicate our previous finding of altered spatial learning and memory in Shank3^E13^ homozygous mutants (Jaramillo et al., 2017).

### Assessing fear conditioning and innate behaviors in *Shank3* mutant mice

**Figure 5.**
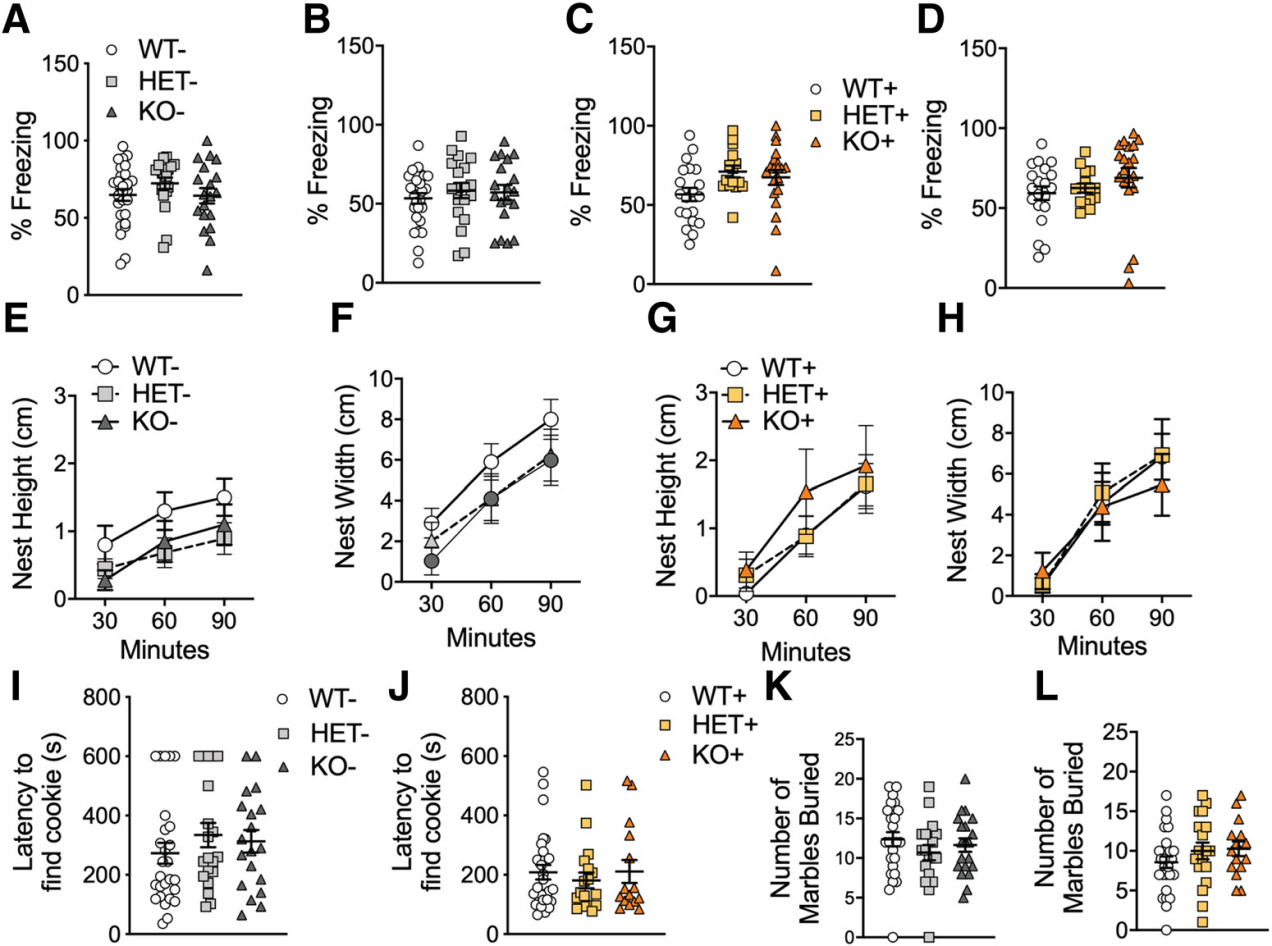
No phenotypes associated with fear conditioning and innate behaviors in *Shank3*– and *Shank3*+ mice. In both the cue (***A***) and contextual (***B***) fear conditioning test, control mice spent similar percent freezing. Similarly, *Shank3*+ mice displayed similar percent freezing in cue (***C***) and contextual (***D***) fear conditioning. In nesting behavior *Shank3*– control mice displayed similar nest heights (***E***) and widths (***F***). Similarly, *Shank3*+ mice displayed similar nest heights (***G***) and widths (***H***). In the palatable treat-finding test, there were no difference observed between *Shank3*– mice groups (***I***) and *Shank3*+ mice groups (***J***), respectively. Additionally, *Shank3*– mice buried similar number of marbles among groups (***K***) and well as *Shank3*+ mice groups (***L***). Data represented as mean ± SEM; *p < 0.05; **p < 0.01, ***p < 0.001, ****p < 0.0001; *n* = 24 WT–, *n* = 16 HET–, *n* = 20 KO–, *n* = 24 WT+, *n* = 19 HET+, *n* = 16 KO+.

Studies in ASD have shown some patients display difficulties updating associations between environmental cues and aversive unconditioned stimuli (South et al., 2012). Thus, we used fear conditioning to test mice for abnormalities in associating a stimulus with aversive consequences. In both cue (Fig. 5*A*) and contextual (Fig. 5*B*) fear conditioning tests, WT–, HET–, and KO– mice displayed similar levels of freezing. Similarly, in the genetically reversed *Shank3* mice, WT+, HET+, and KO+ displayed similar levels of freezing in cue (Fig. 5*C*) and contextual (Fig. 5*D*) fear conditioning. This is the first study of fear conditioning in our Shank3^E13^ mice to date.

Next, we assessed innate behaviors such as nest building and food finding. In the nest-building task, the height and width of nests built over a 90-min time period were assessed. Both control (Fig. 5*E*,*F*) and genetically reversed (Fig. 5*G*,*H*) mice displayed similar levels of nest height and width, respectively, among their genotypes. This is the first test of nest building in our Shank3^E13^ mice to date.

In the food finding test, we buried a palatable treat with home bedding and measured their latency to find the hidden treat. WT–, HET–, and KO– mice displayed similar latencies to find a hidden treat (Fig. 5*I*). Similarly, WT+, HET+, and KO+ mice displayed similar latencies to find the treat (Fig. 5*J*).

Lastly, in the marble burying task, *Shank3* WT, HET, and KO mice in both cohorts buried similar numbers of marbles (Fig. 5*K*,*L*). These negative results in marble burying represent a failure to replicate our previous findings in Shank3^E13^ mutants (Jaramillo et al., 2017).

## Discussion

*Shank3* is among the most well-characterized ASD-associated genes. Genetic analysis in ASD patients has shown there are over 40 known mutations throughout the *Shank3* gene that either lead to truncating variants or are predicted to be deleterious (Jiang and Ehlers, 2013; Mameza et al., 2013; Leblond et al., 2014). Previously we showed that targeted disruption of the PDZ domain in the *Shank3* gene in mice led to a number of behavioral abnormalities (Jaramillo et al., 2017). Mutant mice displayed repetitive grooming, social deficits, rotarod deficits, decrease rearing activity, and learning and memory deficits. These findings validated many findings in a previously published, similar *Shank3* mouse model (Peça et al., 2011).

It is important to note that our genetic approach, based on previous studies (Chen et al., 1998) using this NSE-tTA transgene, was initially expected to limit tTA expression largely to the striatum and cerebellum. Unfortunately, our data revealed a much more widespread NSE-tTA expression based on rescue of our Shank3^E13^ mutant (Fig. 1). We are currently unable to explain this discrepancy other than to suggest that promotor-driven transgenes can vary in their expression based on genetic background, and action of cre may well vary depending on the floxed gene of interest, in this case Shank3^E13^. Second, based on many published reports including our own (Monteggia et al., 2004), we anticipated that breeding and rearing mice on doxycycline-containing water would successfully suppress tTA activation of cre-recombinase expression so that we might use doxycycline withdrawal to regulate the temporal onset of genetic reversal by cre-recombinase. Our data using doxycycline revealed that the cre-recombinase expression was leaky and allowed for genetic reversal of Shank3^E13^ to WT *Shank3* even when doxycycline was provided (data not shown). Thus, we were only able to examine early developmental genetic rescue using this strategy.

Thus, we set out to address the possibility that early genetic restoration of *Shank3* may prevent the onset of the ASD-like behaviors. In combination with two other transgenic mouse lines (tetO-Cre and NSE-TTA), we generated a novel Shank3^E13^ mutant mouse model in which a floxed, transcriptional stop cassette inserted into intron 12 of the *Shank3* gene was removed early in development. We replicated many, but not all, previously published behavioral abnormalities in the control cohort, and observed restoration of SHANK3 expression and reversal of most of the replicated behavioral differences in Shank3^E13^ mice following early genetic reversal.

Mutant KO– mice did not display preference for social novelty, while genetically reversed KO+ mice displayed appropriate preference for social novelty in the three-box social interaction test. Similarly, mutant HET– and KO– mice failed to demonstrate social recognition memory, while both genetically reversed HET+ and KO+ mice displayed normal social recognition memory.

In the genotype/sex-matched test of reciprocal social interaction, HET– mice showed a significant decrease in social interaction compared with WT–, partially replicating our previous findings (Jaramillo et al., 2017). Early genetic reversal of Shank3^E13^ mutation ameliorated this difference in social interaction in the HET+ mice.

In one additional social domain task, we failed to replicate our previously published deficits in mutant KO– cohorts. The caged conspecific social approach test (also called social interaction with a caged adult) did not reveal differences among HET– or KO– and WT–, whereas previously we observed a decrease in the homozygous mutants (Jaramillo et al., 2017). This finding underscores the critical importance of running parallel control cohorts with genetic or pharmacologic reversal experiments. Not doing so may lead to a lack of significant difference being falsely interpreted as rescue of a previously demonstrated behavioral deficit.

We also demonstrated successful genetic rescue of both increased grooming and decreased rearing in our genetically reversible *Shank3* mice. Overall, most behavioral abnormalities demonstrated in our mutant control cohorts were successfully rescued with genetic reversal.

While conducting our study, another group successfully generated and published a similar, genetically reversible *Shank3* mutant model involving adult restoration of *Shank3* in a *Shank3* mutant mouse and showed rescue of social deficits and repetitive grooming behavior in homozygous *Shank3* mutant mice (Mei et al., 2016). Mei et al., also showed the inability to rescue anxiety and motor deficits in adult *Shank3* KO mice. While we did see a similar decrease in the locomotor activity in Shank3+ KO mice in our study, it was apparently only rescued in the heterozygous Shank3*E13* mice. While both studies observed a rescue of ASD-like behaviors, the studies differ on the time points of genetic reversal (Feng et al.: P20–P21 and 2–4.5 months; this study: E18). Both studies replicate genetic reversibility of *Shank3* deficits across similar tests and should be viewed as complementary. However, our study behaviorally characterized genetic rescue of the more clinically accurate heterozygous mutant mice. Following restoration of *Shank3* expression Shank3+ heterozygous mice displayed a lack of repetitive grooming behavior and rescue of social interaction with a juvenile mouse but not a rescue of social preference or social novelty in the three-box social interaction test. The discrepancy in outcomes in social test is likely due to fundamental differences in what each test measures; social interaction with a juvenile mouse in an open arena is a test of reciprocal social interaction whereas the other two tests measure preference for one caged social versus an inanimate or novel target. Also, the age of the stimulus mouse and accessibility of the stimulus mouse differs among the two tests.

Overall, our studies suggest early genetic rescue as a potential genetic therapy for ASD-like behaviors in ASD associated with *SHANK3* deletion/mutation. Taken together with previously published studies, genetic intervention in *SHANK3*-related ASD may be most effective earlier in development.

## Synthesis

Reviewing Editor: Alfonso Represa, INSERM Decisions are customarily a result of the Reviewing Editor and the peer reviewers coming together and discussing their recommendations until a consensus is reached. When revisions are invited, a fact-based synthesis statement explaining their decision and outlining what is needed to prepare a revision will be listed below. The following reviewer(s) agreed to reveal their identity: Jill Silverman, Thomas BOURGERON. Note: If this manuscript was transferred from JNeurosci and a decision was made to accept the manuscript without peer review, a brief statement to this effect will instead be what is listed below.

Both referees appreciated the manuscript and the interest of your results. Both indicated that studies were well performed and addressed an important question about the reversibility of the phenotypes in Shank3 mutant mice. Nevertheless, the paper would highly benefit from a more detailed introduction about the type of restorations that has been conducted, in comparison with existing studies. Indeed, since Shank3 mRNA seems to be almost no detectable before E18, this study actually restores the expression at the same time Shank3 starts to be expressed. The results are still interesting, but clarifying the objectives of the study and the “added value” compared to other studies would improve the interest of this comprehensive characterization. This aspect can also be discussed in the discussion section (paragraph from line 547 on).

There are some concerns about the NSE driver, that was displaying apparently a more widespread expression than that reported before. This point wasn’t discussed and requires clarification. Also this must be taken into account for the interpretation of data.

There was an habituation period for Self-grooming task?

The description of Fear conditioning methods must be improved (duration of the test shock intensity, intervals for recall...)

Concerning figures, Individual points should be added on the bar-plots to provide an insight on the distribution of data.

Concerning the rearing activity during exploration, one option would be that animals had some motor weakness and referees ask for including here “rotarod “data (grip strength or wire hang

The discussion would benefit to be broader, setting this study in the whole literature about the various Shank3 mouse models already existing. For example, the social interaction deficit observed in this Shank3 mutant model was not always observed (Drapeau et al. eNeuro 2018). The discussion (line 556-558) about heterozygous Shank3 mouse could be more discussed since an heterozygous mouse model with a robust phenotype would be of high interest for therapeutic trials.

Also take into consideration the following points:

in the restricted repetitive behaviors section, the word “time’ is missing in the sentence about increase in grooming TIME and number of grooming bouts

In the spatial learning section, Autism et al. 2012 is not cited properly.

l. 541: is “lest” a typo?

l. 225-227: The formulation is strange, as it seems that both the experimental and target mice are two different mice placed together with a juvenile mouse.

l. 261-264: What is the light intensity during grooming measurements?

l. 287-290: It should be stated whether mice were habituated to this palatable food before the test and if all of them ate it.

l. 302-302: The abbreviations E13-/- and E13+/+ have been exchanged.

The title might be more precise than just “many” behaviors.

Figure 2: I suggest to keep the K and L panels with the same scale.

Figure titles are in general not informative and should be edited to provide reader more information.

• Figure 2 should be rearranged to be cleared. A-D should be in one row, then E-G, then H-J and K can be split between two rows. Clearer for reader

• Mean +/- SEM not listed... how is data represented?

• In general, I thought that graphs with the same metric, just either the + or - group, should be on the same axes. So, reader can see the comparison better.

• Figure 5. discrepancy in legend location in H. Should be in G.
